# 3D and Multimodal X‐Ray Microscopy Reveals the Impact of Voids in CIGS Solar Cells

**DOI:** 10.1002/advs.202301873

**Published:** 2023-11-27

**Authors:** Giovanni Fevola, Christina Ossig, Mariana Verezhak, Jan Garrevoet, Harvey L. Guthrey, Martin Seyrich, Dennis Brückner, Johannes Hagemann, Frank Seiboth, Andreas Schropp, Gerald Falkenberg, Peter S. Jørgensen, Azat Slyamov, Zoltan I. Balogh, Christian Strelow, Tobias Kipp, Alf Mews, Christian G. Schroer, Shiro Nishiwaki, Romain Carron, Jens W. Andreasen, Michael E. Stuckelberger

**Affiliations:** ^1^ Center for X‐ray and Nano Science CXNS Deutsches Elektronen‐Synchrotron DESY Notkestr. 85 22607 Hamburg Germany; ^2^ Fachbereich Physik Universität Hamburg Luruper Chaussee 149 22761 Hamburg Germany; ^3^ Paul Scherrer Institute PSI Forschungsstrasse 111 Villigen 5232 Switzerland; ^4^ Deutsches Elektronen‐Synchrotron DESY Notkestr. 85 22607 Hamburg Germany; ^5^ National Renewable Energy Laboratory 16253 Denver West Parkway Golden CO 80401 USA; ^6^ Helmholtz Imaging Deutsches Elektronen‐Synchrotron DESY Notkestr. 85 22607 Hamburg Germany; ^7^ Department of Energy Conversion and Storage Technical University of Denmark DTU Fysikvej 310 Kongens Lyngby 2800 Denmark; ^8^ DTU Nanolab Technical University of Denmark DTU Ørsteds Plads 347 Kongens Lyngby 2800 Denmark; ^9^ Institut für Physikalische Chemie Universität Hamburg Grindelallee 117 20146 Hamburg Germany; ^10^ Laboratory for Thin Films and Photovoltaics Empa Ueberlandstrasse 129 Dübendorf 8600 Switzerland

**Keywords:** CIGS, multimodal X‐ray imaging, nano‐tomography, ptychography, thin film solar cells

## Abstract

Small voids in the absorber layer of thin‐film solar cells are generally suspected to impair photovoltaic performance. They have been studied on Cu(In,Ga)Se_2_ cells with conventional laboratory techniques, albeit limited to surface characterization and often affected by sample‐preparation artifacts. Here, synchrotron imaging is performed on a fully operational as‐deposited solar cell containing a few tens of voids. By measuring operando current and X‐ray excited optical luminescence, the local electrical and optical performance in the proximity of the voids are estimated, and via ptychographic tomography, the depth in the absorber of the voids is quantified. Besides, the complex network of material‐deficit structures between the absorber and the top electrode is highlighted. Despite certain local impairments, the massive presence of voids in the absorber suggests they only have a limited detrimental impact on performance.

## Graphical abstract

3D X‐ray microscopy quantifies the distribution of voids in thin‐film solar cells and associated electrical performance deficits.

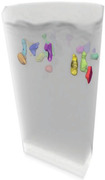

## 1 Introduction

Thin film solar cells with a CuIn_x_Ga_(1‐x)_Se_2_ (CIGS) chalcopyrite absorber offer a promising and commercially viable alternative to Si‐wafer‐based solar cells with potential for cost‐effective single‐junction and tandem solar cells.^[^ ^1^, ^2^, ^3^, ^4^ ^]^ The progress of this technology, achieving efficiency >23%,^[^ ^5^ ^]^ was made possible by two main breakthroughs: a proper Ga–In grading profile and a heavy‐Alkali postdeposition treatment.^[^ ^6^ ^]^ At Empa, such steps were implemented in a multi‐stage process^[^ ^7^ ^]^ that, however, introduced voids in the top region of the absorber layer, close to the CdS buffer layer. In fact, the formation of voids and pinholes is a relatively common shortcoming, reported for various polycrystalline thin‐film absorbers.^[^ ^8^, ^9^, ^10^, ^11^, ^12^ ^]^

To increase the cost‐efficiency of the cells and bridge the efficiency gap between cells and modules, recombination losses must be reduced. Besides undermining the structural integrity of the device, voids reduce contact area and are suspected to host recombination centers.^[^ ^13^ ^]^ With respect to performance, Avancini et al. assessed their effect as detrimental through simulations.^[^ ^14^ ^]^ In their investigation and the others, single voids were imaged in lateral cross sections by transmission electron microscopy and energy dispersive spectroscopy, and a top view of the absorber was obtained by uncovering the upper layers through focus ion beam (FIB) milling. However, the drawbacks of FIB milling are to inevitably enlarge the voids of interest and to prevent further analysis by destroying the sample.

In this paper, we build upon these previous investigations by applying synchrotron X‐ray imaging to elucidate the nature of these structural defects. Exploiting multimodal scanning X‐ray microscopy, we have demonstrated the ability to map areas of up to several hundred square microns, measuring electrical performance through X‐ray beam‐induced current and voltage (XBIC^[^ ^15^ ^]^ and XBIV^[^ ^16^ ^]^), optical performance via X‐ray excited optical luminescence (XEOL^[^ ^17^ ^]^), elemental composition through X‐ray fluorescence (XRF), and electron area‐density via ptychography.^[^ ^18^ ^]^ This approach is established for top view^[^ ^17^ ^]^ and cross sections^[^ ^19^ ^]^ of a layer stack, but still only in 2D. However, voids are known to extend along a certain depth of the absorber and it is desirable to visualize them along all dimensions. Ptychographic X‐ray computed tomography (PXCT)^[^ ^20^, ^21^ ^]^ appears well suited for this purpose, based on previous results on other thin‐film solar cells,^[^ ^22^ ^]^ enabling nondestructive quantitative 3D imaging. State‐of‐the‐art beamlines can currently measure a tomogram of a volume in the order of 10 µm^3^ at 20 nm resolution in a matter of hours.

## 2 Experimental Section

**Figure 1:**
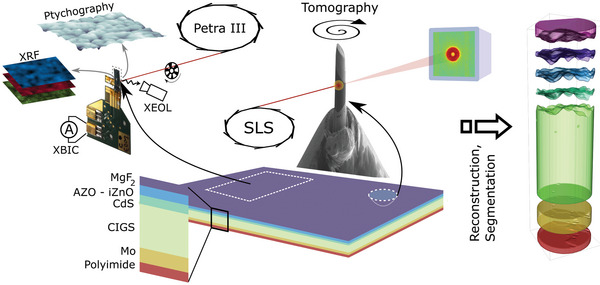
Experimental concept. Two samples of the same layer stack are prepared for two different experiments. In the first experiment, a sample is mounted on a printed circuit board and electrically contacted so that maps of the induced current can be measured. In the second experiment, the sample is carved out and mounted on a pin, where it is molded to a cylindrical shape. Projections from different angles are acquired to yield a 3D reconstruction that can be processed and decomposed into individual layers.

To investigate the effect of nano‐voids on performance, samples for two different synchrotron experiments were prepared. The experimental concept and setup are illustrated in **Figure** **1**. The layer stack of the samples under investigation comprises from top to bottom: a MgF_2_ antireflective coating (105 nm); Al:ZnO top contact layer (65 nm); ZnO window layer (120 nm); CdS buffer layer (20–50 nm); CIGS absorber (≈3 µm); Mo rear contact layers (500 nm); and a polyimide substrate. The samples were taken from a cell whose fabrication process resulted in a 20.2% efficiency for its best cell.^[^ ^7^ ^]^ Details about the cell are available in the Supporting Information. In the first multimodal measurement, an area sized ca. 5 × 5 µm^2^ was raster‐scanned at the microprobe of PETRA‐III beamline P06 (DESY)^[^ ^23^ ^]^ at an energy of 15.25 keV, slightly above the absorption edge of Rb. Compound refractive lenses were used with a phase plate to focus the coherent beam to a 105 nm spot size.^[^ ^24^ ^]^ The different techniques were performed in three successive scans and scan parameters were differently optimized for ptychography and XEOL, respectively, and registered for the analysis. For the second experiment, a pillar of 5 µm diameter covering the entire layer stack was isolated through FIB, and a PXCT scan was performed at the SLS beamline cSAXS (PSI)^[^ ^25^ ^]^ at an energy of 6.2 keV on the flOMNI setup.^[^ ^26^ ^]^ Whereas the multimodal scans provide a single top view of the sample, tomography provides the full 3D image that can be represented by vertical or horizontal slices and can be further processed to independently analyze the single layers. Both for 2D and 3D images, the ultimate goal was to label pixels and voxels that present evidence of material deficit, and therefore refer to voids, i.e., volumes of thin films characterized by absence of material.^[^ ^14^ ^]^

## 3 Results and Discussion

### 3.1 Multimodal 2D X‐ray Imaging

**Figure 2:**
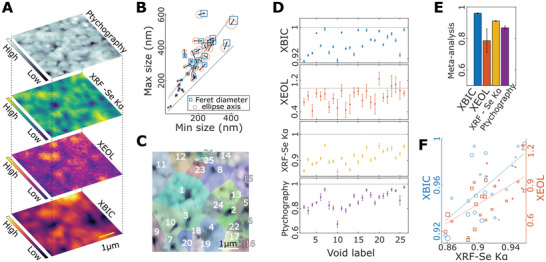
Multimodal 2D X‐ray imaging of CIGS solar cell. A) Stack of top‐view multimodal maps. Ptychography and fluorescence maps (top), locating voids in the absorber; XEOL and XBIC (bottom) mapping their optical and electrical performance. B) Extent of voids estimated from ellipse‐fitting or Feret diameters. Fitting dashed lines relate to the eccentricity of the voids. C) Labels of voids and surroundings segmented from the XRF map and represented in overlay transparency on the ptychography map. D) Relative measurements of XBIC, XEOL, XRF, and ptychography within labeled voids. Expressed as the ratio between average intensity within void and outside void. E) Average loss and extent of voids resulting from a meta‐analysis of measurement in (D). F) Scatter plot of XBIC and XEOL versus Se fluorescence counts. Dashed lines indicate a linear trend of increasing performance per increasing XRF counts. The size of markers in (B) and (F) is proportional to the void area.

Some of the maps obtained through different modalities are reported in **Figure** **2**A. Voids are identified both through fluorescence and ptychography. Among all fluorescence signals, the most reliable and statistically relevant for this purpose is arguably the Se Kα, because of the stoichiometry of CIGS including at least twice as many atoms as the other elements and because possible secondary phases are all likely to contain Se. Nonetheless, the presence of voids can also be well noted in the Cu and Ga Kα maps (Figure S1, Supporting Information). Unlike fluorescence, ptychography does not have elemental sensitivity but provides quantitative maps of the electron density. Moreover, ptychography can have a resolution that is not limited by the beam size and achieves in our case the higher resolution (30 nm estimated via Fourier ring correlation,^[^ ^27^ ^]^ Figure S2, Supporting Information) that resolves crevices between CIGS grains. Due to these features, fluorescence and ptychography provide pictures of voids in the absorber layer, respectively, before and after the deposition of the top layers. However, these are known from previous investigations to be strongly correlated,^[^ ^17^ ^]^ to the extent that ultimately the ptychography map and the Se map describe essentially the same features, i.e., local material deficits and the morphology of the grains in the absorber layer. The latter is responsible for a larger length scale variation that makes it more appropriate to analyze single voids with respect to their own surroundings, i.e., the sets of nearest neighboring pixels. The average size of the segmented voids is estimated with fitting ellipses or Feret diameters as ≈300 nm but exhibits considerable variation (Figure 2B). In Figure 2D we report statistics extracted from the 26 largest voids segmented from the Se map. For each void labeled in Figure 2C, Figure 2D shows the ratio of the measurements averaged over pixels within a void and the measurements averaged over its surroundings. The XRF values fall below one by definition and the deviation from one relates to the material deficit of a void or the porosity of the filling. The ptychography values are strongly correlated to XRF values, and for this reason, statistics extracted from the segmentation of voids in XRF and ptychography do not differ substantially (cf. Figure S3, Supporting Information). In fact, scanning electron microscopy (SEM) cross‐sections from previous investigations have shown that the layers deposited on the absorber mostly follow the morphology of the absorber, which leads to assuming that a top‐view image of the stack essentially depicts the absorber. Later in this work, we use tomography to validate this assumption.

More importantly, both the relative XBIC and XEOL measurements appear almost exclusively below one, indicating and quantifying a local performance impairment. These techniques image competing processes, i.e., they count, respectively, generated electron‐hole pairs that diffuse to the electrodes without recombining, and photons with an energy corresponding to the bandgap emitted upon radiative recombination. XBIC appears less impaired by voids than XEOL. The larger error bars from XEOL are due to the nature of the tracked process, i.e., XEOL is intrinsically a photon‐hungry technique. Performance is locally impaired up to 8% for XBIC and 60% for XEOL. On average, the effect of voids can be quantified by the meta‐analysis (see Supporting Information for details) in a 4% loss for XBIC and 20% for XEOL (Figure 2E). XEOL, in particular, degrades more than expected from the missing absorber, which might be an indication that voids and crevices are detrimental to cell voltage. This impairment can be attributed to the amount of missing material or an enhanced recombination velocity at interfaces with voids.^[^ ^28^, ^29^ ^]^ However, it is not trivial to decouple these effects, as the performance maps are effectively blurred by the diffusion length of the carriers and by the electron shower produced by the beam (see Supporting Information). Such blurring leads to an underestimation of the XBIC dips, which explains why on most voids the relative reduction of XRF exceeds its XBIC counterpart. Despite the underestimation, the measured impairment is consistent and significant, and given the high overall performance of the device, suggests that charge transport in the functioning device is mostly sustained by alternative current paths than those below the worst performing areas. The optical and electrical performance losses correlate positively with the amount of missing material (Figure 2F) (correlation coefficients ≈0.7), whereas they do not correlate, if not weakly, with the projected area of the voids.

The local performance impairment observed through measurements of current and luminescence cannot be as clearly observed when induced by a laser beam or an electron beam. In the case of lasers, the fundamentally limited resolution does not allow us to investigate the topic, as even the largest voids are smaller than the diffraction limit of photoluminescence measurements.^[^ ^30^ ^]^ In the case of electrons, the investigation of the effect of voids is hampered by internal scattering effects. Our complementary measurements of EBIC and CL show high‐signal spikes in the proximity of voids (see Figures S12 and S13, Supporting Information). These spikes are not an indication of enhanced electrical or optical performance due to specific material properties, but rather a measurement artifact due to secondary electrons being reabsorbed (see Supporting Information). Whereas these scattering phenomena are negligible for X‐rays, voids can have positive effects under AM1.5G illumination due to light trapping.^[^ ^31^ ^]^ A minor performance impairment at voids is predicted by 3D numerical simulations of simple exemplary cases, although without accounting for optical effects.^[^ ^14^ ^]^

### 3.2 Ptychographic Nanotomography

**Figure 3:**
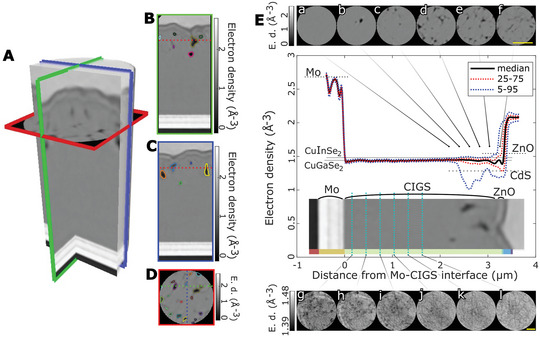
Depth information from PXCT of CIGS solar cell. A) Volume rendering of cylindrical sub‐volume with electron density in greyscale. The diameter is 2 µm. B–D) Sagittal, coronal, and axial slices. Segmented voids are highlighted by colored contours E) Depth profile of electron density across the layer stack. Blue and red lines indicate the 5–95 and the 25–75 percentile variations. Most variation occurs within 500 nm below the CdS layer. Axial slices a–f) from the upper region of the CIGS layer are reported above. Slices g–l) from the lower part of the absorber, represented with truncated greyscale to enhance contrast. Scale bars are 1 µm.

Whereas the 2D top‐view of the sample highlights lateral differences in the operational device, it cannot provide depth information about the disclosed features. Effectively, they can be vertical projections of multiple features, in the same or different layers. In fact, it is of interest to locate their depth, as due to the Ga–In grading^[^ ^32^ ^]^ and the vertical inhomogeneity of the CdS layer, voids can have a different impact depending on their depth in the stack. Ptychographic tomography can elucidate such features. The technique uses a set of coherent diffractive scanning projections from different angles and phase‐retrieval algorithms to map in 3D the complex refractive index of the interaction volume, whose real part δ is proportional to the electron density and whose imaginary part β is proportional to the mass absorption coefficient (see Supporting Information for details). The technique is today renowned for its astounding resolution and quantitativeness.^[^ ^33^ ^]^ Some exemplary cuts and a volume rendering of the device under investigation are shown in **Figure** **3**. The δ‐tomogram shows clearly the layer stack and the voids. Spatial resolution for the δ‐tomogram was assessed in the 30–40 nm range (see Figure S4, Supporting Information), with the standard deviation of measured electron densities being below 2% of the average measurement (see Figure S6, Supporting Information). The edge profile across voids and interfaces (Figure S4, Supporting Information) decays roughly within the same distance for δ‐ and β‐tomograms, whereas uncertainty is larger in β‐tomograms and artifacts are more severe. Quantitative values of electron density extracted from δ reveal the profile distribution of Figure 3E, which locates the voids in the upper part of the absorber, ≈500 nm below the buffer‐absorber interface. The electron‐density distribution is more uniform in the lower part of the cell, except for a slight rise in the direction of the top electrode, which is due to the Ga grading^[^ ^34^ ^]^ and is better visible in the β‐tomogram (Figure S5, Supporting Information). Moreover, very small voids are visible at the bottom of the absorber (Figure 3E, slices g‐i), which are not expected to be detrimental^[^ ^31^ ^]^ and likely correspond to the nucleation sites of CIGS grains. Finally, lower electron‐density traits can be noticed at mid‐height (Figure 3E, slices k,l), which we may identify as grain boundaries, based on the expected grain size. It is not possible to ascertain whether the contrast for these traits is provided by a gap between grains, or by Na or Rb selenides, among which RbInSe_2_ is likely.^[^ ^35^, ^36^ ^]^ Other secondary phases, such as Cu‐or Mo‐selenides, which would be discernible by electron‐density contrast, are not present in the device.

**Figure 4:**
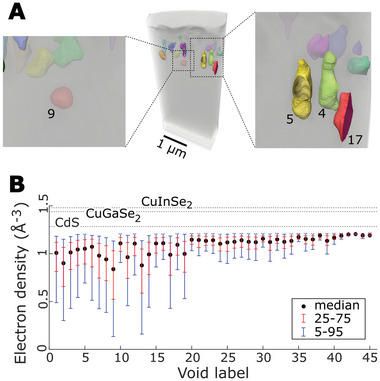
3D segmentation and analysis of voids. A) Volume rendering of the PXCT data with segmented voids. The left inset depicts a spherical deep void, right inset depicts a group of vertically elongated voids B) Electron density within the single voids. Blue and red lines indicate the 5–95 percentile and the 25–75 percentile variations.

The voids were segmented in an inner 2 µm‐diameter cylinder unaffected by sample preparation artifacts with a threshold‐and‐watershed algorithm as 45 labeled regions with a spatially confined material deficit (**Figure** **4**A). The segmentation parameters were set to exclude any nonporous components of the CIGS‐CdS interface region. In particular, the threshold value was set below the expected electron density of the lightest element material (CdS), taking measurement uncertainty into account. Moreover, this segmentation excludes very small voids that are within the lower part of the CIGS and voids that are below resolution (3‐voxel size) but includes sets of voxels that are partly void and partly filled by CdS, as in spots of imperfect adherence. The voids were then fit by ellipsoids to analyze their size and orientation (Figures S7–S9, Supporting Information). This analysis shows that there is no evident correlation between height and volume of the voids. Figure 4B illustrates the electron‐density distribution within the void regions, which possibly relates to the CdS filling for the largest voids, but is affected also by partially filled voxels and blurred edges in the smallest material‐deficit regions. Orthogonal views and renderings of the single voids are reported in Figure S10 (Supporting Information). Notable examples are voids nine and 13 which are likely occluded from the top and are not reached by the CdS chemical bath deposition (cf. Figure 4B; Figure S10a,b, Supporting Information). No particular shape is detected except for a few almost spherical voids (e.g., 9,19). The general picture supported by the statistics of Figure S7 (Supporting Information) is that of voids with sizes between 100 and 400 nm, mostly elongated in the vertical direction, and generally with low convexity.

**Figure 5:**
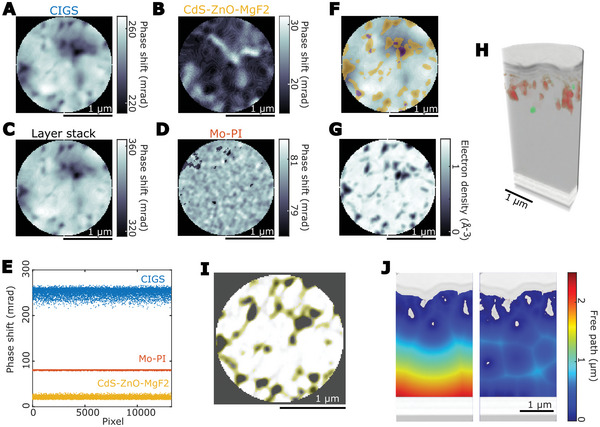
Comparison of 2D versus 3D ptychography. A–D) Vertical projections as computed from tomography of the CIGS layer, CdS‐ZnO‐MgF_2_, layer stack, and Mo‐polyimide (PI). E) Dispersion of values within layers illustrated in A, B, D. F) Map of overlapping voids at different heights. Areas in yellow and purple cross one and two voids respectively. G) Minimum intensity projection of the absorber. H) Volume rendering of voids as isolated (green) and connected to the buffer layer (red). I) The network of crevices connecting the voids (color highlight, slice view). J) Two hypothetical scenarios of free path distance for charge carriers across the absorber, based on measured electron density values. Void surfaces lie mostly at the large voids (left) or they lie across the whole stack (right), i.e., across unpassivated grain boundaries and minor voids.

Besides the voids, we segmented every single layer of the device stack (see Supporting Information for details), which resulted in the exploded view displayed in Figure 1. This allows us to verify and quantify the assumption that a 2D map of the cell is mostly representative of the absorber. The top view projection of the full stack and groups of layers is depicted in **Figure** **5**A–D. There, we note that the other layers (including highly scattering Mo) show their own structure and features and that the dispersion in the absorber (Figure 5E) is higher than in the other layers altogether (σ_CIGS_ = 8 mrad vs σ_rest_ = 3 mrad). Consequently, the stack projection predominantly follows variations of the absorber, with the correlation coefficient between the CIGS projection and the full stack projection being R = 0.95, and the correlation coefficient between the CIGS projection and that of the stack of layers above CIGS being the second largest R = −0.67. Such a large negative value can be attributed to an overall effective filling of voids within the chemical bath deposition of CdS. Besides, the absorptance of each layer above CIGS is negligible compared to that of CIGS (Figure S14, Supporting Information). Within this comparison of 2D and 3D data, we also note that regardless of a certain degree of arbitrariness involved in the choice of segmentation parameters, the void segmentation for 2D and 3D data contains notable differences. Whether segmented using the Se or the ptychography map, the voids cover an area of ≈6% of the multimodal maps vs ≈25% of the 2D projection extrapolated from the tomogram (Figure 5F). A similar disproportion is observed for the density of single voids. The reasons can be ascribed to a better intrinsic sensitivity to the void edges provided by 3D data, the inaccurate grouping of distinct overlapping voids as single, and the inability to discriminate between regions of CdS and topographical variation. The improved clarity of features obtainable in 3D is also illustrated by a minimum intensity projection (Figure 5G), which is a multiplanar image of the lowest‐density features along the projection axis. Moreover, this type of projection (cf. Figure S11, Supporting Information) emphasizes the numerosity of the voids, whose area density appears significantly higher than previously reported.^[^ ^14^ ^]^

Along with better sensitivity to edges, the tomogram slices show the crevices between grain boundaries in the top part of the absorber (Figure 5I), which are likely filled by CdS. These features are partly visible in 2D (Figure S2, Supporting Information) and in 3D show that they form a network of voids in which most but not all are connected (Figure 5H). Whereas all voids originate from the crevices of the polycrystal, the ones that form at a lower depth are more likely to be enclosed and not be reached by the CdS. Numerical simulations show that for the same surface recombination velocity, a buried void is slightly less detrimental than an interface void.^[^ ^14^ ^]^ As Rb tends to segregate at grain boundaries,^[^ ^35^, ^37^, ^38^ ^]^ whether CdS locally forms a p‐n junction or not, determines whether downward or upward band‐bending occurs, hence causing a detrimental or beneficial effect for charge carrier transport.^[^ ^39^ ^]^ In our case, only a minor downward bending and a moderate recombination velocity at the interface are expected, based on more recent measurements of PL and charge carrier lifetimes from cells of the same process flow with similar performance^[^ ^30^ ^]^ (see Figure S16, Supporting Information).

The network of voids is of critical importance for performance as it contains pathways for the diffusion of impurities, possibly leading to interface recombination. Recent developments for high‐energy X‐ray focusing,^[^ ^23^ ^]^ resonant ptychographic tomography, and correlative 3D microscopy^[^ ^40^, ^41^ ^]^ can further reveal the extent to which this network is filled by CdS and impurities, and will enable the unambiguous distinction between Cd and In to determine whether p–n junctions are locally formed. Whether only the large void regions or the whole network with deep small voids are considered within the absorber, the two scenarios of the distance map in Figure 5J can be drawn (see Figure S6C, Supporting Information). These maps yield an average free path well below the diffusion length,^[^ ^34^ ^]^ 0.6 versus 3 µm in the worst case, which, along with the good cell performance, suggests that most of the voids are effectively passivated and supports the model of preferential current paths within CIGS grain.^[^ ^42^ ^]^

## 4 Conclusion and Outlook

Altogether, we have shown in this study the 3D nature of structural defects in thin‐film CIGS solar cells and we identified local performance deficits attributable to voids. Although possibly detrimental at a local level, the high density of voids highlighted by tomography suggests that their effect cannot be dramatic at the device level, given the high efficiency of the cell. We point out that such a complex system is not easily modeled and available finite element simulation results are not directly comparable with our measurements.^[^ ^14^ ^]^ Our measurements with absolute electron densities quantified at the nanoscale enable the development of adequate models simulating structural and electronic defects.^[^ ^19^ ^]^ This investigation does not alter the void size or shape as FIB‐SEM might do, however, it does require a state‐of‐the‐art X‐ray microscopy beamline and involves a delicate sample preparation. Future experiments should aim to avoid it, probe larger areas, and explore the sample in a multi‐scale approach. Such goals may be achieved with a laminography setup.^[^ ^43^ ^]^ Regarding the availability of beamtime, accepting a loss of resolution, similar studies can be extended to the best lab‐CT instruments. Other thin film solar cells, perovskites before all, in single‐junction or tandem configuration, demand studies of this kind to elucidate fabrication defects and improve cost‐efficiency.

In general, our study highlights the sensitivity at the nanoscale to a multitude of physical, chemical, and electrical properties, enabled by synchrotron imaging. These results are particularly timely in view of novel scanning X‐ray microscopes that are under development and will become operational in the coming years, in which the full set of techniques of the multimodal toolset may be performed at the same time and in 3D. The possibility of such simultaneous measurements can in turn foster future in situ and operando studies of growth, performance, and degradation,^[^ ^44^, ^45^ ^]^ which can help bridge the gap between cell and module efficiency. The enhanced brilliance of fourth‐generation sources^[^ ^46^ ^]^ will overcome limitations of sample size and scan duration for the 3D case.

## Conflict of Interest

The authors declare no conflict of interest.

## Author Contributions

M.E.S.: Conceptualization; G.Fe, C.O., M.V.: Data Curation; M.E.S., G.Fe.: Methodology; R.C., S.N., M.V., H.L.G., J.G., G.Fa., A.Sc., F.S., C.G.S., C.S., T.K., A.M., Z.I.B., J.W.A, M.E.S.: Resources; G.Fe., C.O., M.V., H.G., M.S., A.Sc., M.E.S.: Visualization; A.M., G.Fa., C.G.S., J.W.A., M.E.S.: Funding acquisition; G.Fe., C.O., H.L.G., A.Sl., P.S.J., M.V., C.S., D.B., J.H., A.Sc., F.S., T.K., J.W.A., M.E.S.: Investigation; G.Fe., C.O., M.V., M.E.S. : Formal Analysis; M.E.S.: Project administration; M.S.: Software; A.M., C.G.S., T.K., J.W.A., M.E.S.: Supervision; G.Fe., C.O., M.V., H.L.G., J.H., G.Fa., R.C., J.W.A., M.E.S.: Validation; G.Fe., M.E.S.: Writing—original draft; G.Fe, C.O., M.V., J.H., G.Fa., R.C., J.W.A., M.E.S.: Writing—review and editing.

## Supporting information

Supporting Information

Supplemental Movie 1

Supplemental Movie 2

## Data Availability

The data that support the findings of this study are available at the open‐access repository https://doi.org/10.5281/zenodo.10018968. Raw data are available from the corresponding authors upon reasonable request.
