# Supplementary material for: 3D and Multimodal X‐Ray Microscopy Reveals the Impact of Voids in CIGS Solar Cells
Source: Adv Sci (Weinh). 2023 Nov 27;11(2):2301873. doi: 10.1002/advs.202301873 (PMC10787091; doi:10.1002/advs.202301873)
Supplement: Supplementary file 1 — Supporting Information [file ADVS-11-2301873-s001.pdf]

ADVANCED SCIENCE
Open Access

Supporting Information

for *Adv. Sci.*, DOI 10.1002/advs.202301873

3D and Multimodal X-Ray Microscopy Reveals the Impact of Voids in CIGS Solar Cells

Giovanni Fevola, Christina Ossig, Mariana Verezhak, Jan Garrevoet, Harvey L. Guthrey, Martin Seyrich, Dennis Brückner, Johannes Hagemann, Frank Seiboth, Andreas Schropp, Gerald Falkenberg, Peter S. Jørgensen, Azat Slyamov, Zoltan I. Balogh, Christian Strelow, Tobias Kipp, Alf Mews, Christian G. Schroer, Shiro Nishiwaki, Romain Carron, Jens W. Andreasen and Michael E. Stuckelberger**

Supplementary Materials for

Three-dimensional and multimodal X-ray microscopy reveals the impact of voids in CIGS solar cells

Authors: G. Fevola[1*], C. Ossig[1,2], M. Verezhak[3], J. Garrevoet[4], H. L. Guthrey[5], M. Seyrich[1], D. Brückner[4], J. Hagemann[1,6], F. Seiboth[1], A. Schropp[1], G. Falkenberg[4], P. S. Jorgensen[7], A. Slyamov[7], Z. I. Balogh[8], C. Strelow[9], T. Kipp[9], A. Mews[9], C. G. Schroer[1,3,6], S. Nishiwaki[10], R. Carron[10], J. W. Andreasen[7], M. E. Stuckelberger[1]*

* Correspondence to giovanni.fevola@desy.de & michael.stuckelberger@desy.de.

This PDF file includes:

Materials and Methods
Supplementary Text
Figs. S1 to S10
Tables S1 to S2
Captions for Movies S1 to S2

Other Supplementary Materials for this manuscript include the following:

Movies S1 to S2
References and Notes

Materials and Methods

Materials and device fabrication

The $Cu(In,Ga)Se_2$ (CIGS) cell was synthetized at Empa within the process 'D' detailed in (*7*). In this process a 500 nm Mo back contact layer was deposited by sputtering on a polyimide substrate. The CIGS absorber was deposited in a multistage co-evaporation process at 450 ºC substrate temperature and underwent an in-situ NaF+RbF post-deposition treatment in Se ambient. The final Ga:In composition ration is 0.41:0.59 and the absorber thickness is 3.16 um. Followed chemical bath deposition of the CdS buffer layer (25 nm); sputtering deposition of intrinsic ZnO (65 nm) and of Al:ZnO (110 nm). The cell was completed with a 105 nm MgF_2 anti reflective coating.

Photovoltaic performance

The photovoltaic performance parameters of the best cell of this process are: efficiency 20.2%; open-circuit voltage Voc=715.0 mV; short-circuit current Jsc=36.7 mA/cm^2;
fill factor FF=77.0%. The box plots of Figure S15 summarize the parameters of 18 sister cells of the same process.
In the discussion on CdS-CIGS interface in the main text, we refer to PL measurements on devices coming from the same institute, with the same device architecture and process flow (*30*). However, the authors of ref. (*30*). studied the effect of different substrates and deposition temperatures, which resulted in varied performance that is, overall, comparable to our device. For details, we refer to Table 1 of ref. (*30*).

Multimodal X-ray Imaging

The x-ray microscopy setup used for the multimodal imaging experiment is described in detail in (*17*), section 2.2.
The measurements reported in the main article result from three scans, optimized respectively for: 1) XBIC, 2) XEOL, 3) ptychography. XRF was recorded for all the scan and it was used to register the resulting maps in post-processing. The XRF maps reported in the main text and used to compute void statistics are from the XEOL scan. The scan parameters are summarized in Table S1.
For the XBIC scan the cell was short circuited and measured according to the established protocol detailed in (*15*). A chopper was used to modulate the beam, which effectively halves the photon flux. XEOL was measured with the cell in open-circuit mode and with a longer dwell time to provide acceptable signal to noise ration. For the ptychography scans the chopper was removed and the slit width was reduced to increase the coherent fraction of the x-ray beam. XEOL was measured with an objective and an optical camera, and it was not spectrally resolved. The total measured counts are almost exclusively due to CIGS, with a negligible contribution from CdS and no contribution from ZnO. In fact, although CdS has absorptance and quantum yield in the same order of magnitude as CIGS, it is much less present in the sample, with a nominal thickness of ca. 1% than CIGS thickness. ZnO emits in the ultraviolet range, which is not detected by our camera.

Ptychographic X-ray Computed Tomography

Ptychographic x-ray computed tomography was performed at the cSAXS beamline of Swiss Light Source (SLS) at the Paul Scherrer Institute, Villigen, Switzerland (*25*). The flOMNI setup was used (*26*). A 6.2 keV x-ray beam, focused by a Fresnel zone plate (*47*), was used to scan the sample. The sample was placed 51.9 mm after the focal spot, where the probe diameter was ca. 3 um.

A field of view of $9 \times 4\ \mu m^2$ was covered by a Fermat spiral pattern with a step step-size of 300 nm. Exposure time was set to 0.1 s per step. Scan duration was ca. 6 hours.

The ptychographic projections were reconstructed with PtychoShelves (*48*), using a 800×800 pixel area of an Eiger (*49*) 4M detector placed 5268 mm downstream from the sample, resulting in a pixel size of 17.6 nm. The reconstruction ran with 300 iterations of the difference map algorithm (*18*) , followed by 2000 iteration of maximum likelihood refinement (*50*).

For tomography, 328 projections equally spaced in a 180-degree angular range were measured. The phase δ and amplitude β of the ptychographic projections were aligned in post-processing, and the constant and linear phase offset were removed from the phase. The δ- and β-tomographic reconstructions were obtained from such projection with a modified filtered back projection algorithm (*51*, *52*).

For the δ-tomogram resolution was assessed with Fourier shell correlation in an interior region of the sample as 37 nm based on the ½ bit criterion (Fig. S4 a,b).

Multiple line profile estimates with the 10-90% criterion across the Mo-PI interface give on average 40 nm and 54 nm for resolution in δ- and β-tomograms, respectively (Fig. S4 c,d).

Meta-analysis of multimodal data

The goal of the meta-analysis is to provide a general synthetic quantifier for the effect of voids. We referred to (*53*) for the method theory and we carried out the analysis similarly to our previous work (*54*). Briefly, each single void is individually studied by comparing it to its immediate surroundings. The void i is characterized by a population of pixels within the void and a population in its surroundings (control group).

We define the effect of void i on measurand M as the ratio between the mean of these two populations:

$$r_i^M = \frac{< M_i >_{void}}{< M_i >_{surrounding}},$$

where $<\cdot>_x$ indicates the average operator over population x.

The standard error E_i^M is used to quantify uncertainty of M with respect to each void:

$$E_i^M = \frac{1}{\sqrt{N_i}} \frac{\sigma_{i,void}^M}{M_i|_{void}} + \frac{1}{\sqrt{N_s}} \frac{\sigma_{i,surrounding}^M}{M_i|_{surrounding}},$$

where σ indicates the standard variation of measurand M and N are population sizes.

Therefore, we evaluate treat each void as a sub-study and evaluate the effect size $\overline{M}_w$ as the weighted average of measurands M:

$$\overline{M}_w = \frac{\sum_{i=1}^k w_i\, r_i^M}{\sum_{i=1}^k w_i},$$

with k being the number of voids and the weights w_i defined as

$$w_i = \frac{1}{(E_i^M)^2 + \sigma^2},$$

and σ^2 being the variance of r_i^M among the k voids.

Analysis ptychographic tomography

Ptychographic tomography resolves a discretized volumetric object in terms of its complex refractive index:

$$n = 1 - \delta - j\beta \quad (1)$$

where j is the imaginary unit. δ and β relate, respectively, to the physical phenomena of scattering and absorption, therefore they can be equivalently expressed in terms of phase shift and absorption coefficient:

$$\phi = \frac{2\pi}{\lambda}\,\delta\,\mathrm{dz}, \quad (2)$$

$$\mu = \frac{4\pi}{\lambda}\beta, \quad (3)$$

where λ is the wavelength of the impinging beam, dz is the voxel size.

Moreover, δ can be further related to another material property, i.e. the electron density of the material, as follows:

$$n_\mathrm{e} = \frac{2\pi\delta}{\lambda^2 r_\mathrm{e}}, \quad (4)$$

where r_e is the classical electron radius. Such relation applies when λ is far from absorption edges of elements included in the object.

The general relation between the refractive object and the material properties of the sample is given by:

$$n(\lambda) = 1 - \delta - j\beta = 1 - \frac{r_\mathrm{e}}{2\pi}\lambda^2 \sum_k n_\mathrm{at}^k\,(f_1^k + jf_2^k), \quad (5)$$

in which f_1^k and f_2^k are real and imaginary atomic scattering factors of the k-th atomic species, and n_at^k its density.

Eq. 5 provides the means to estimate the expected refractive index of a known material system from tabulated values of the atomic factor (*55*). The estimates for materials contained in the layer stack are reported in Table S2.

Uncertainty is estimated as in (*56*), and results are reported in Fig. S6 A,B. Fig. S6C reports the distribution of measured electron densities within segmented layers of the cell, compared to

nominal values of Table S2. The ZnO layer was segmented using the ratio δ/β to enhance contrast with neighboring layers.
The scenarios of Fig. 5J in the main text delve into the assessment of voids, relating to the thresholding method used to identify them, and describe the two most representative cases, in which a higher and a less conservative threshold (blue and green dotted lines in Fig. S6D) are used. Therefore, the scenarios depict only the effect of voids on charge collection due to non-negligible recombination at the void surfaces. These topographical considerations are independent of the band-bending discussion.

Analysis and visualization tools

Watershed segmentation was performed with the Interactive H Watershed in Fiji.
Volume rendering images and video were produced with ThermoFisher Scientific Avizo 11.

Electron beam induced current and cathodoluminescence investigation

In an attempt to further validate the results of the multimodal x-ray imaging, we investigated the sample with electron microscopy. A co-processed sister cell was investigated through top-view SEM, EBIC, and CL. EBIC maps were acquired with an accelerating voltage of 5 kV and an incident beam current of ~1.5 nA.
For CL measurements, the top layers were polished through Ar-ion milling to obtain enough CL signal from the top absorber layer that is highest interest. During the milling, the Ar ions were directed under a small angle to the sample surface to flatten the topography, i.e. to non-selectively eliminate all material above a certain height. Maps as large as 8x8 μm2 were acquired with 15 kV accelerating voltage and an incident beam current of ~2.5 nA.
Standard SEM images, obtained through secondary electrons, were acquired along each EBIC and CL image. All measurements were conducted at room temperature.
As previously observed (*17*), the top-view SEM images of the finished device offer already some insights into the location of voids, as the deposition of the thin layers covering the CIGS absorber are mostly conformal. We know from x-ray tomography that this view is not complete as deeper voids are buried. However, these buried voids are rare – most voids are superficial and extend for up to a few hundred nanometers.
The most representative results of the CL/EBIC investigation are collected in Fig. S12, S13. The maps refer to three different regions of the sample. For each region, the SEM image is shown next to an EBIC image or to a panchromatic CL image. The spectrum of CL (not shown) lies in the expected range for CIGS, but does not show significant variation nor correlation to features such as grain boundaries.
EBIC maps of pristine regions (before milling) are inversely correlated to the SEM images (see Fig. S12 a,b). This can be attributed to topographical contrast induced by the reabsorption of secondary electrons. CL images are not available for pristine samples as the signal intensity was too weak without milling.
The EBIC maps of polished regions (Fig S12 c,d) show large (μm size) dark spots that follow evidently in most cases the shape of a grain (cf. largest dark spot in the insets). These spots of low EBIC may come from originally bad grains or from grains on top of the absorber whose top surface has been removed by milling, leaving surface defects. Both mechanisms would result in an impaired charge collection efficiency.

The other evidence to note in these maps is that some grain boundaries appear darker than the grain cores, indicating a higher recombination rate, which suggests an imperfect passivation of the grain boundaries. These results are compatible with our findings from XBIC and XEOL but do not add significant information.

Note also the bright spots in the center of grain boundary intersections both in EBIC and CL images. These are interpreted again as a topographic absorption effect at voids, where the reabsorption of secondary electrons leads to enhanced signal. The correspondence between locations of voids and spikes is particularly evident in the CL image (Fig. S13).

Moreover, one may speculate that voids contribute to loss mechanisms similarly to grain boundaries. However, our XEOL and CL images do not allow us to validate the similarity. Indeed, a grain boundary recombination velocity can be extracted from the decay of a CL line scan across a grain boundary (*57*) and fed into a two-dimensional numerical model to simulate cell performance (*58*), thus linking electron microscopy measurements to overall device performance. However, the same approach cannot be pursued for grain boundaries lying next to voids, as line scans across the CL spikes of Fig. S12 and S13 do not show an exponential decay as in (*57*). Whereas a decay in proximity of voids is observed in XEOL images, to adequately compare grain-to-grain boundaries to grain-to-void boundaries, contrast methods that are complementary to the electron density are needed, e.g. diffraction based. Future studies based on x-ray nano-diffraction may shine further light on the grain-to-void-boundary relation, exploiting the reduced reabsorption of X-rays compared to electrons.

In fact, the x-ray approach mitigates reabsorption in XEOL through a fundamentally different interaction with matter compared to electrons in CL. In the case of electron-based CL measurements, the cascade from high-energy electrons to excited electron-hole pairs is dominated by electron scattering, and many electrons escape the sample. Accordingly, topographic features of the sample such as voids have a strong impact on the reabsorption of escaped electrons. In the case of x-ray based XEOL measurements, the cascade from high-energy photons to excited electron-hole pairs is initiated by photoabsorption that takes place comparably deep in the bulk after straight propagation of the x-ray photons (x-ray scattering is negligible at this scale). Scattering of electrons only occurs as a second-order effect and further away from the surface. Accordingly, fewer electrons escape and topographic features of the sample have a smaller impact on the XEOL signal.

<u>Note on CIGS band-bending</u>

Whereas only downward bending is expected at CdS-CIGS interfaces, it is known that grain-to-grain interfaces can induce downward or upward bending (*39*).

The indication of only moderate band bending for our cells comes from the experimental observation of a quasi-single-exponential decay of the time resolved- photoluminescence (TR-PL) signal, as in the right part of Fig. S16 from ref. (*30*). This is only compatible with a small surface recombination velocity and small band-bending as evidenced by the simulations shown in the left part of Fig. S16.

Furthermore, a strong band bending and high recombination velocities, whether at interfaces between CIGS and CdS or at CIGS grain boundaries (unfilled voids), would have a very detrimental impact on TR-PL lifetime, PL emission, and device performance (mostly V_{oc}), which has not been observed in the devices that undergo alkali post-deposition treatments in reference (*30*) and in our study.

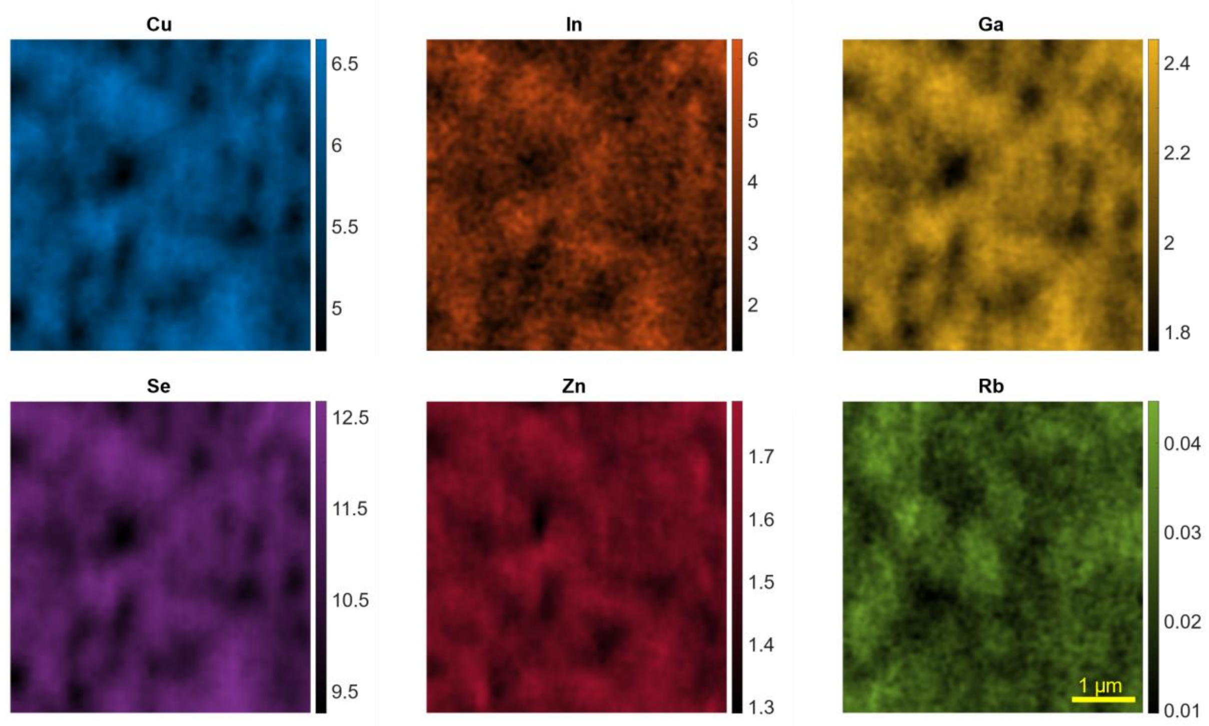

Fig. S1. Elemental area density maps from multimodal imaging, XRF.

Units of colorbars are µmol/cm^2 and scale bar is common to all maps. All maps are extracted from K lines except for the In map, which is extracted from L lines and appears noisier for this reason.

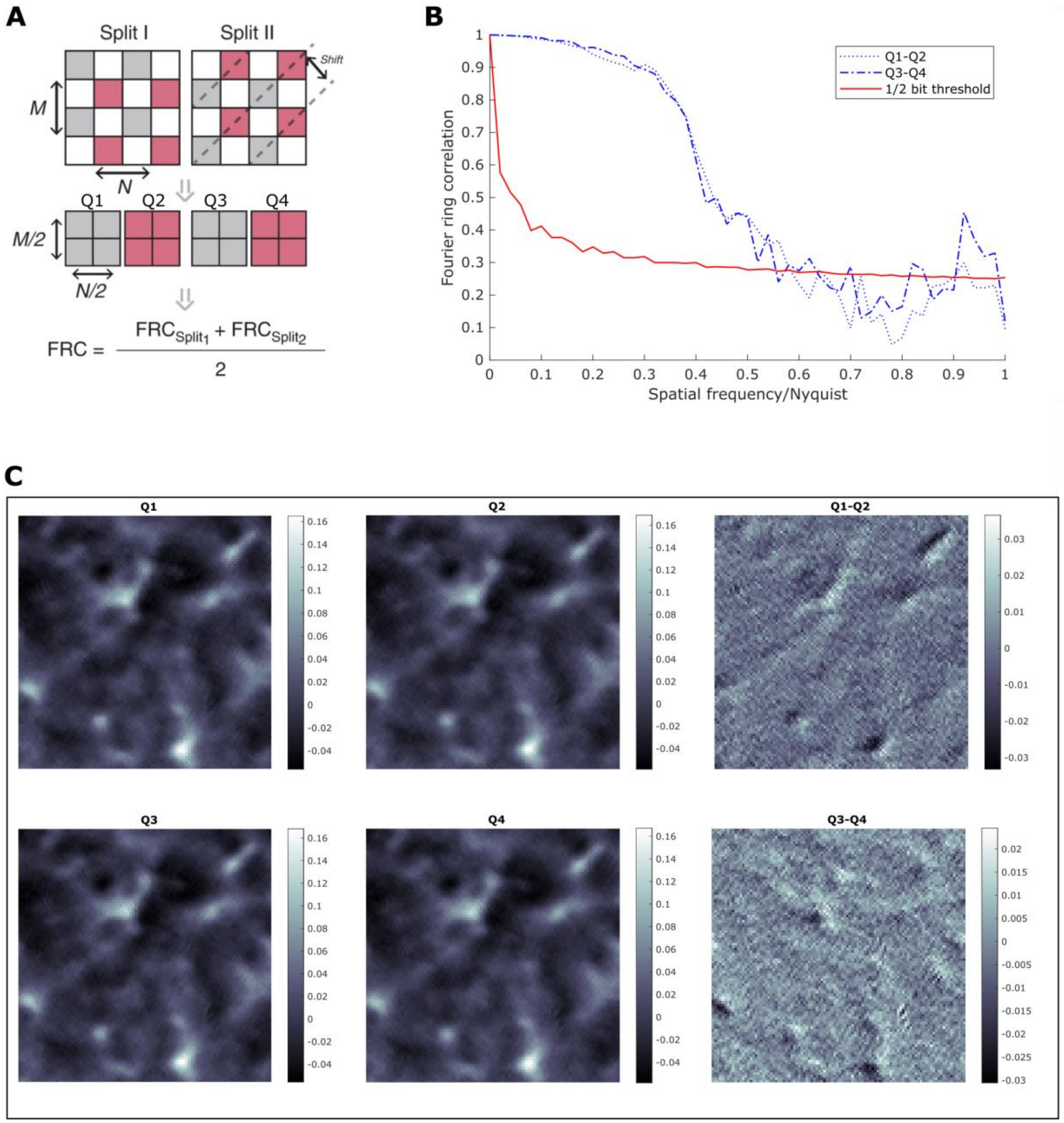

Fig. S2. Resolution of 2D ptychography. (A) Scheme for one-image FRC, adapted and reproduced with permission from (*59*). **(B)** Fourier ring correlation values for ptychography map of multimodal experiment. **(C)** Binned images and their difference.

Intercept values for the two splits are 29.2 and 30.7 nm, which yields 30.0 nm average. Maps in C are also meant to illustrate visibility of crevices in 2D.

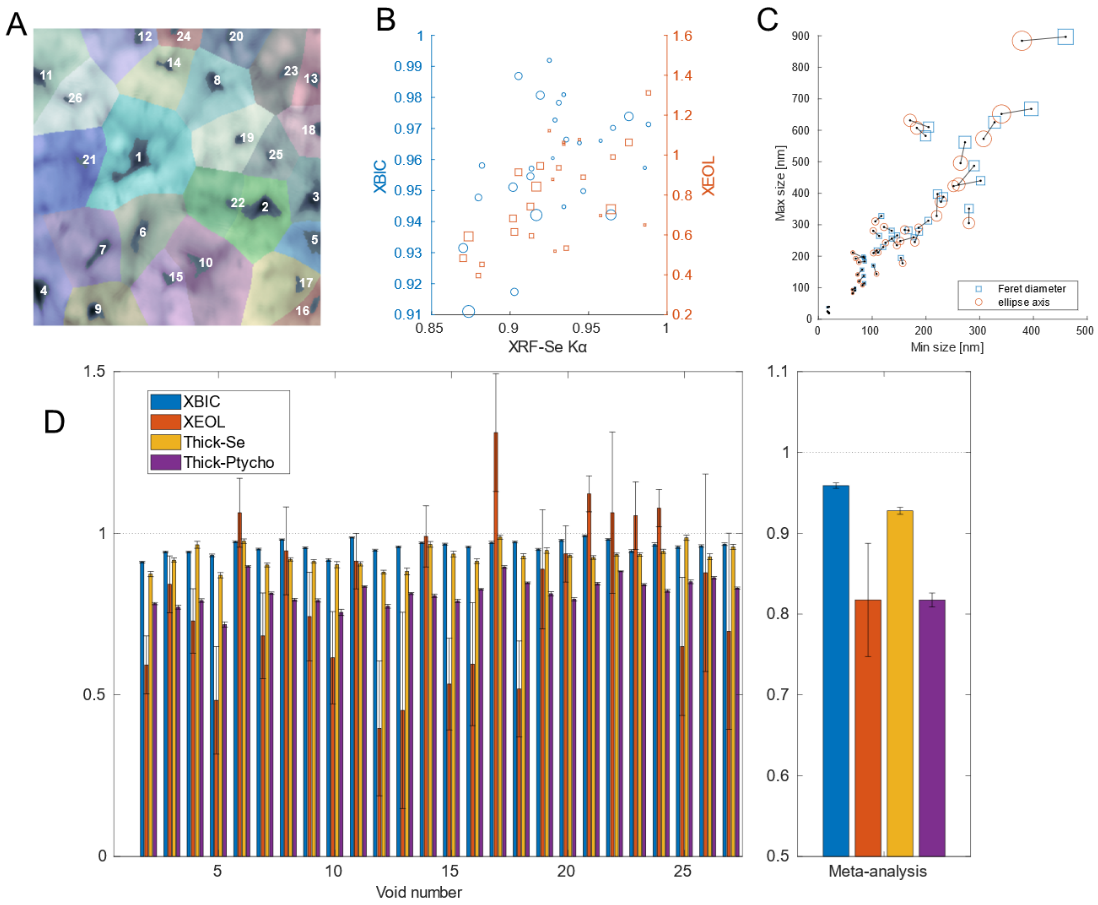

Fig. S3. Meta-analysis of voids segmented from ptychography map.
Same analysis as in Fig. 2 main text, except that here voids are segmented from the ptychography map.

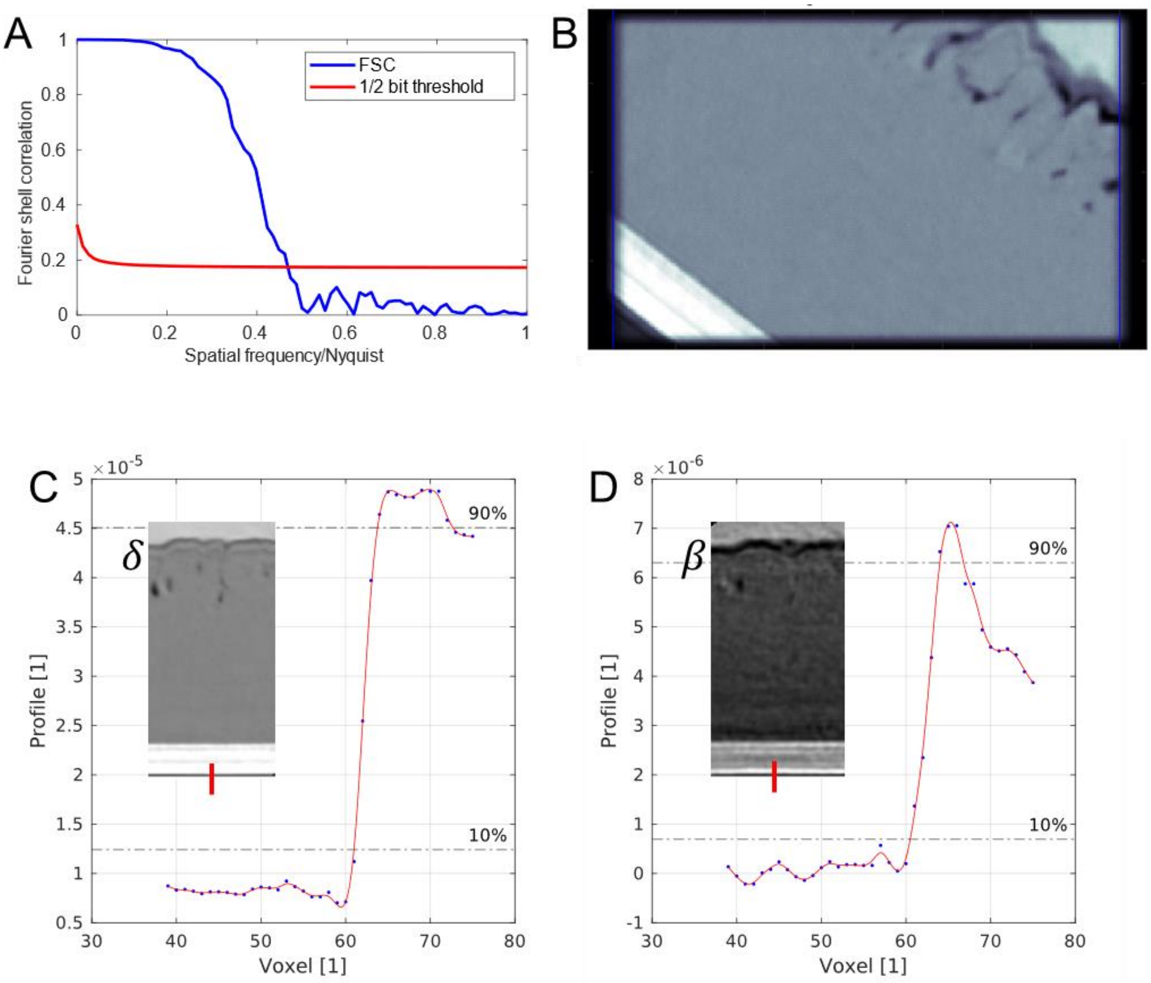

Fig. S4. Resolution of ptychographic tomography. (A) Fourier shell correlation curve. The threshold is intercepted at 37 nm. **(B)** Slice of volume selection for FSC. **(C, D)** Edge profiles across Polyimide-Mo edge (red line) for δ- and β- tomogram respectively, which give ~3 and ~3.5 voxels resolution according to the 10-90% criterion.

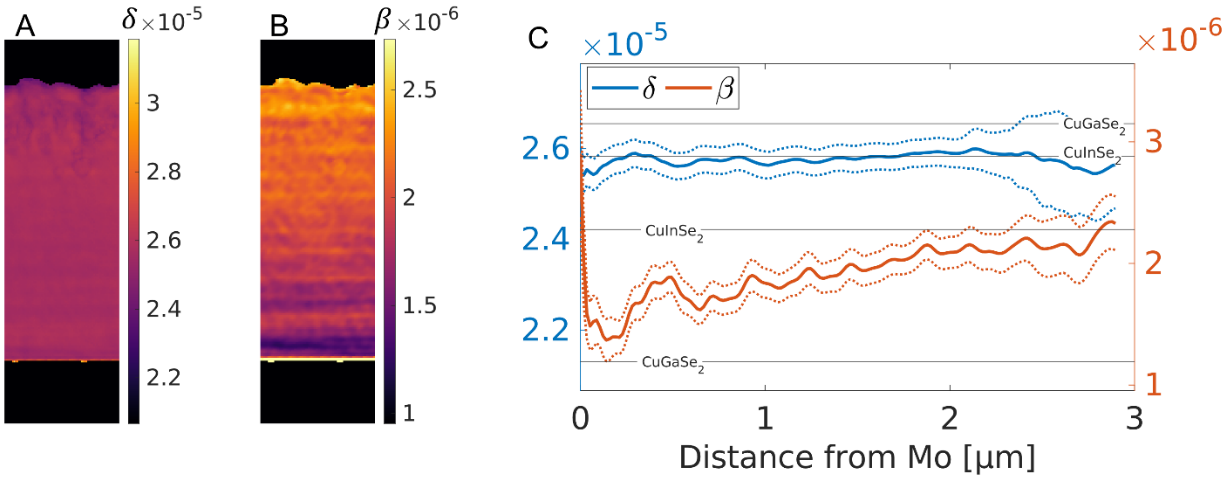

Fig. S5. Ga-In contrast in CIGS. (A, B) Laterally averaged δ and β. Width of images is 2 μm. **(C)** Profile along depth of values averaged within slice. Voids are excluded in this analysis.

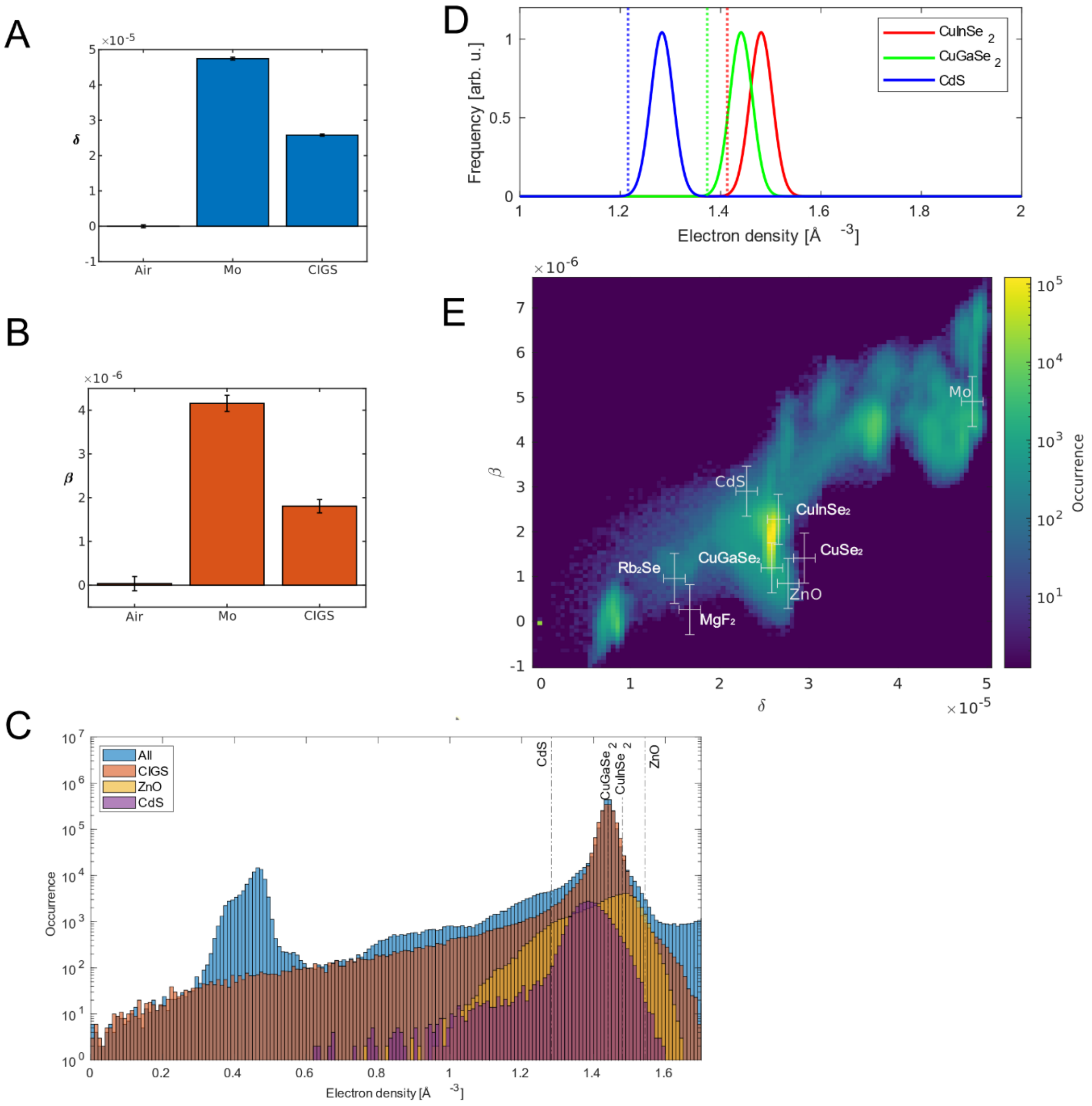

Fig. S6. Uncertainty quantification in ptychographic tomography. (A, B) δ and β within volumes of air, Mo, and CIGS. Bar length and uncertainty are mean and standard deviation of the distribution. **(C)** Histogram of voxels within tomogram. It is illustrated how the expected values of CIGS matches the measurements. The two peaks referring to ZnO and CdS are distinct but under- and over- estimated, respectively. **(D)** Scheme for thresholding. The Gaussians represent the expected distribution of CIS, CGS, and CdS. Their variance is based on estimates from measurements and a coverage factor 3 is used. **(E)** Colocalization plot. For ZnO, δ/β provides enhanced contrast and was exploited for segmentation.

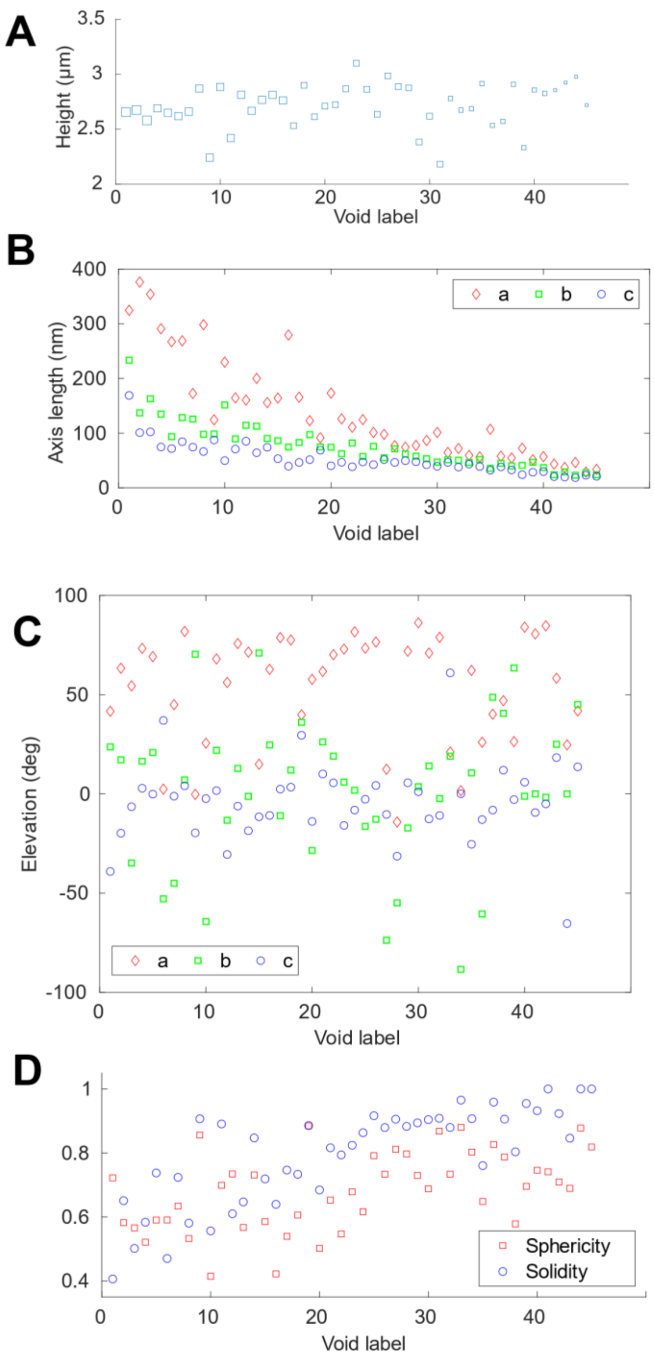

Fig. S7. Statistics of voids. (A) Height of voids in the absorber, expressed as distance from Mo. **(B, C)** Length and orientation of principal axes of ellipsoids fitting the segmented voids. The axes are denoted as $\mathbf{a} \geq \mathbf{b} \geq \mathbf{c}$. **(D)** Shape and convexity statistics. Sphericity is here defined as $\mathbf{c}^2/(\mathbf{ab})$ and solidity as the ratio of the volume of the void and that of the smallest convex hull that contains it. Labels in plots are sorted by volume.

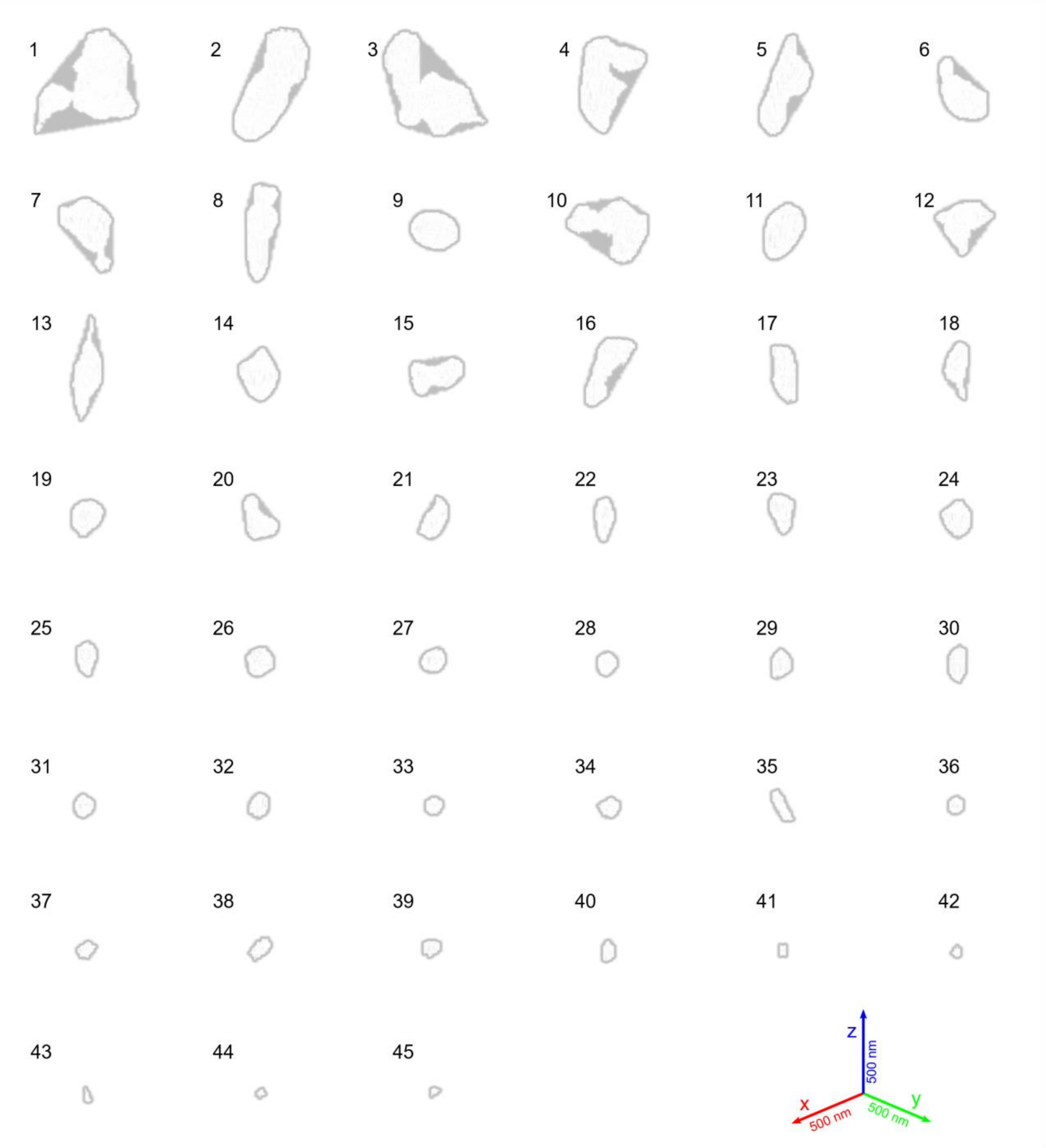

Fig. S8. 3D rendering of voids highlighting solidity, shape, size.

The analyzed voids are represented in their convex hull. The grey contours delineate the smallest convex hull containing a void, whose image is the bright area inside.

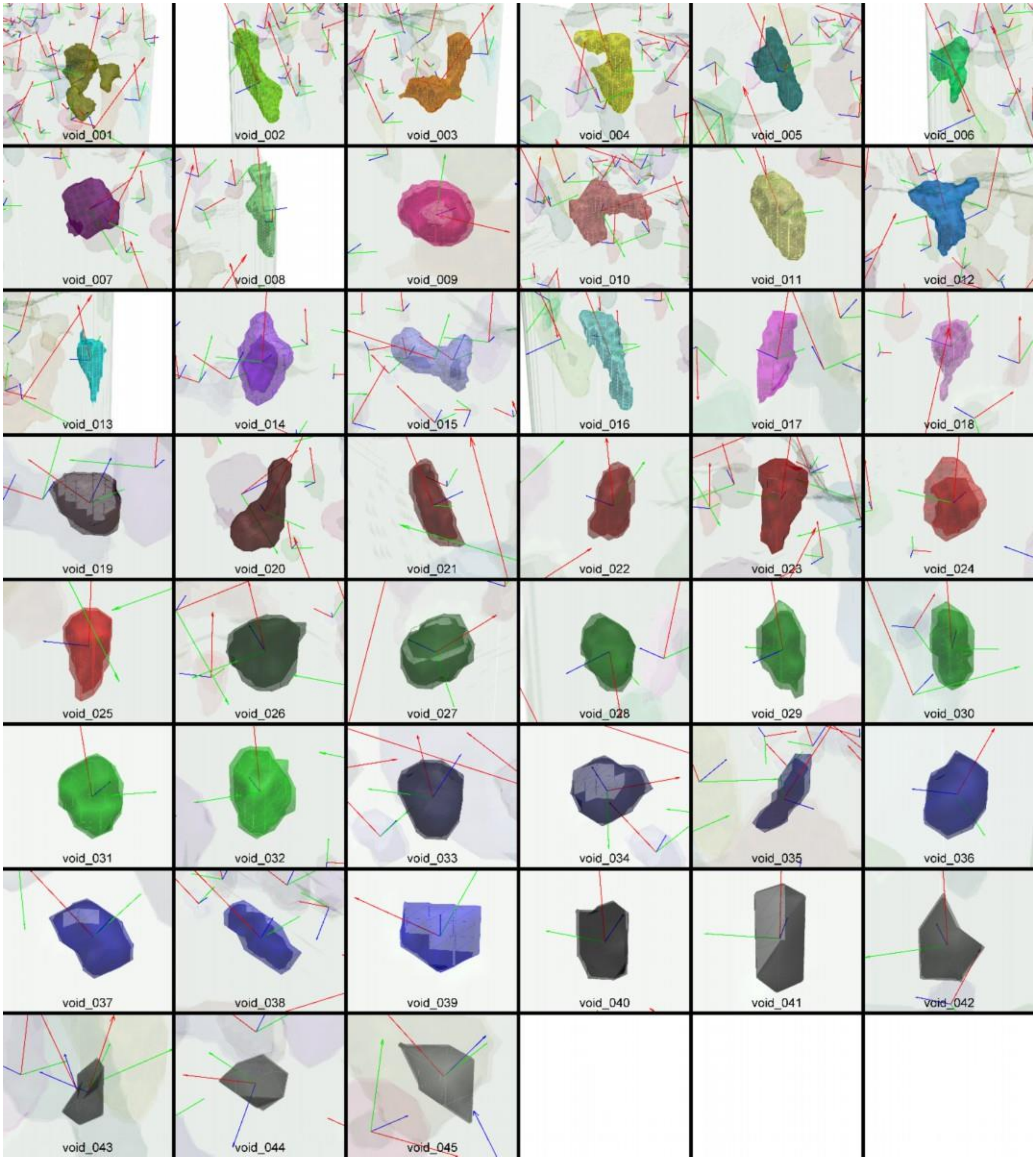

Fig. S9. Rendering orientation.

Refer to Movie S2 to see the full animated view.

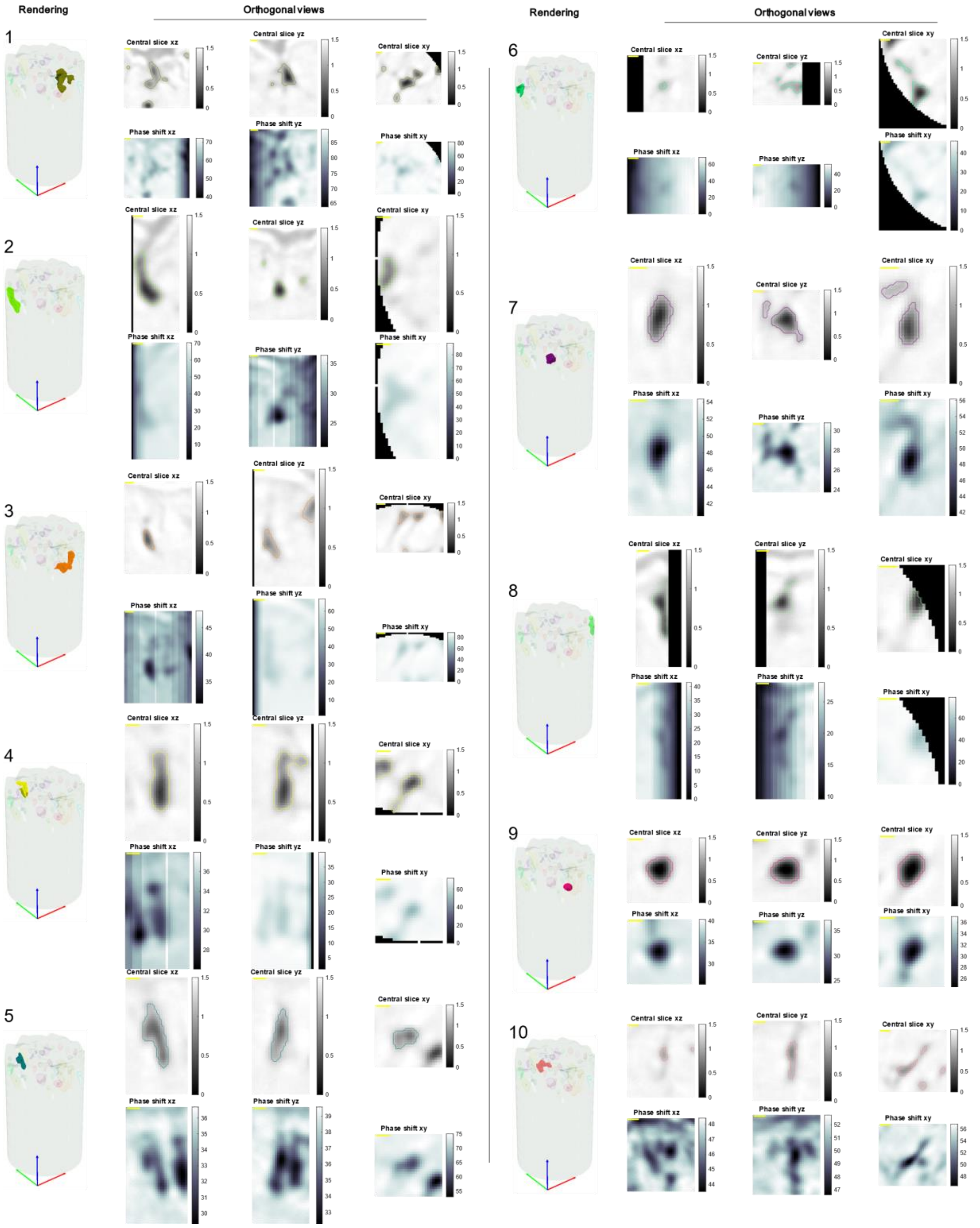

Fig. S10-a. View of single voids.

Each void is represented by label and rendering, orthogonal central slice including segmentation contours (top 3 images, 'gray' colormap) and projection (phase shifts, bottom 3 images, 'bones' colormap). Scale bars are 100 nm, units are electron density for the slices and mrad for phase shifts.

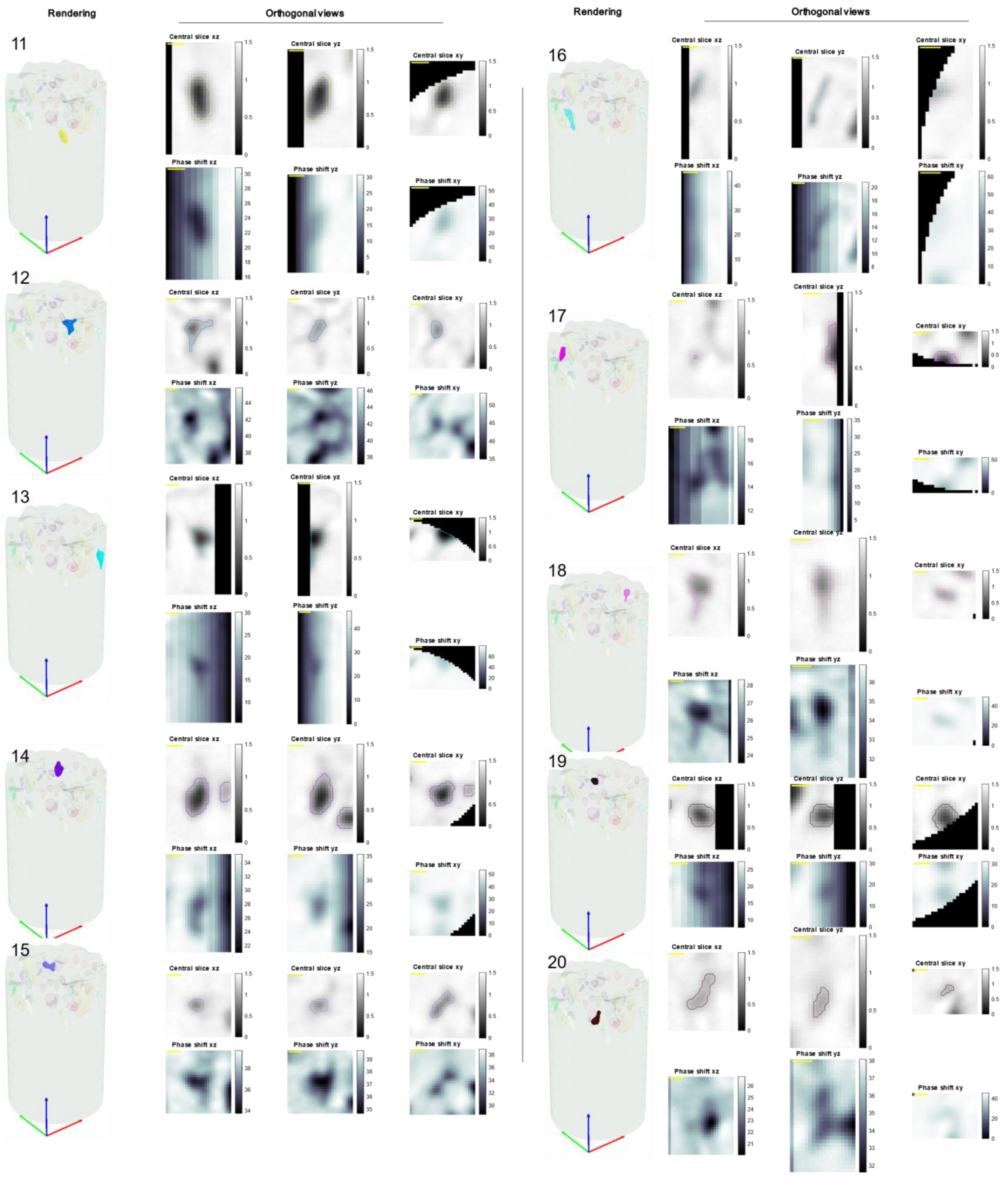

Fig. S10-b. View of single voids.

Each void is represented by label and rendering, orthogonal central slice including segmentation contours (top 3 images, ‘gray’ colormap) and projection (phase shifts, bottom 3 images, ‘bones’ colormap). Scale bars are 100 nm, units are electron density for the slices and mrad for phase shifts.

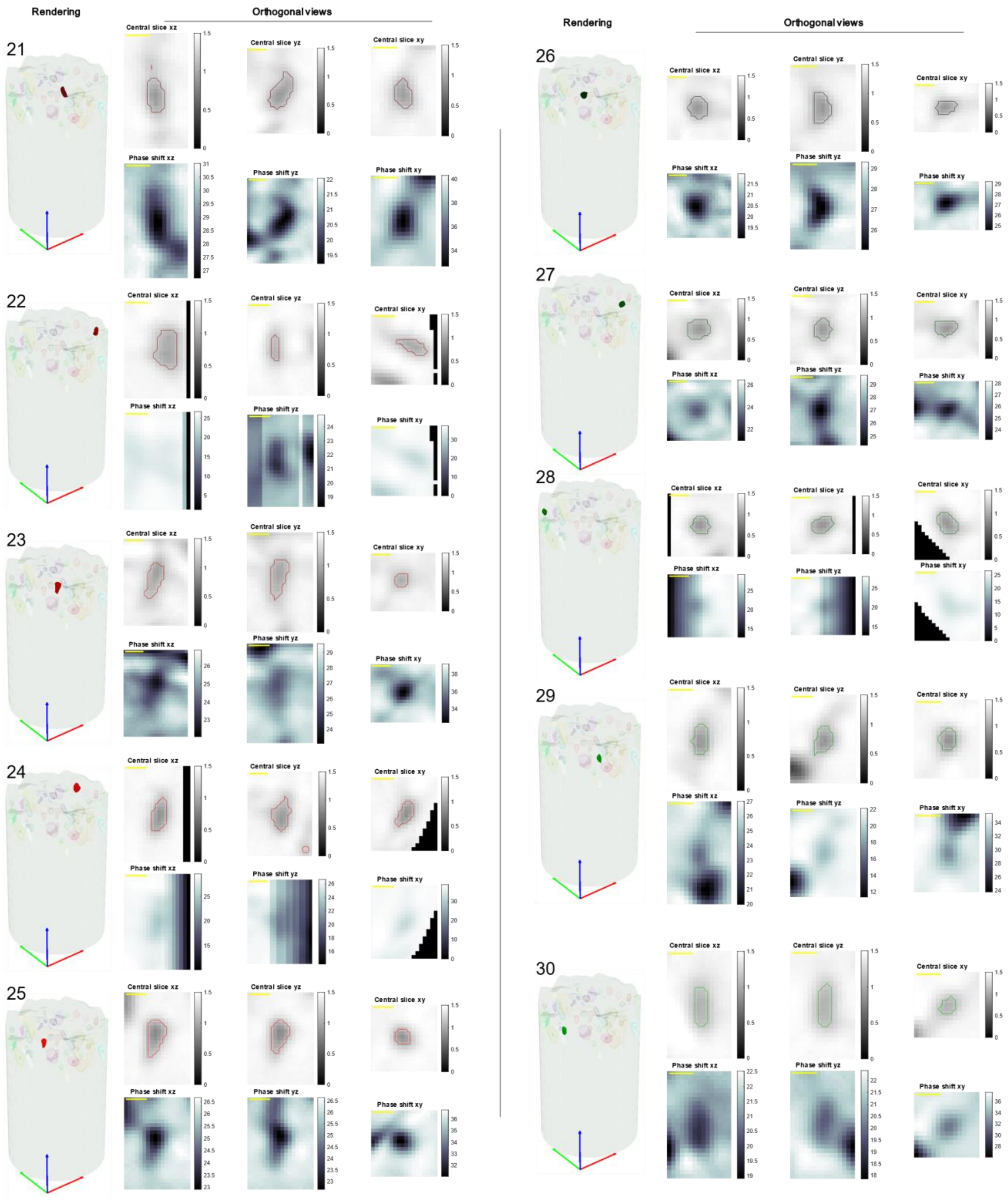

Fig. S10-c. View of single voids.

Each void is represented by label and rendering, orthogonal central slice including segmentation contours (top 3 images, ‘gray’ colormap) and projection (phase shifts, bottom 3 images, ‘bones’ colormap). Scale bars are 100 nm, units are electron density for the slices and mrad for phase shifts.

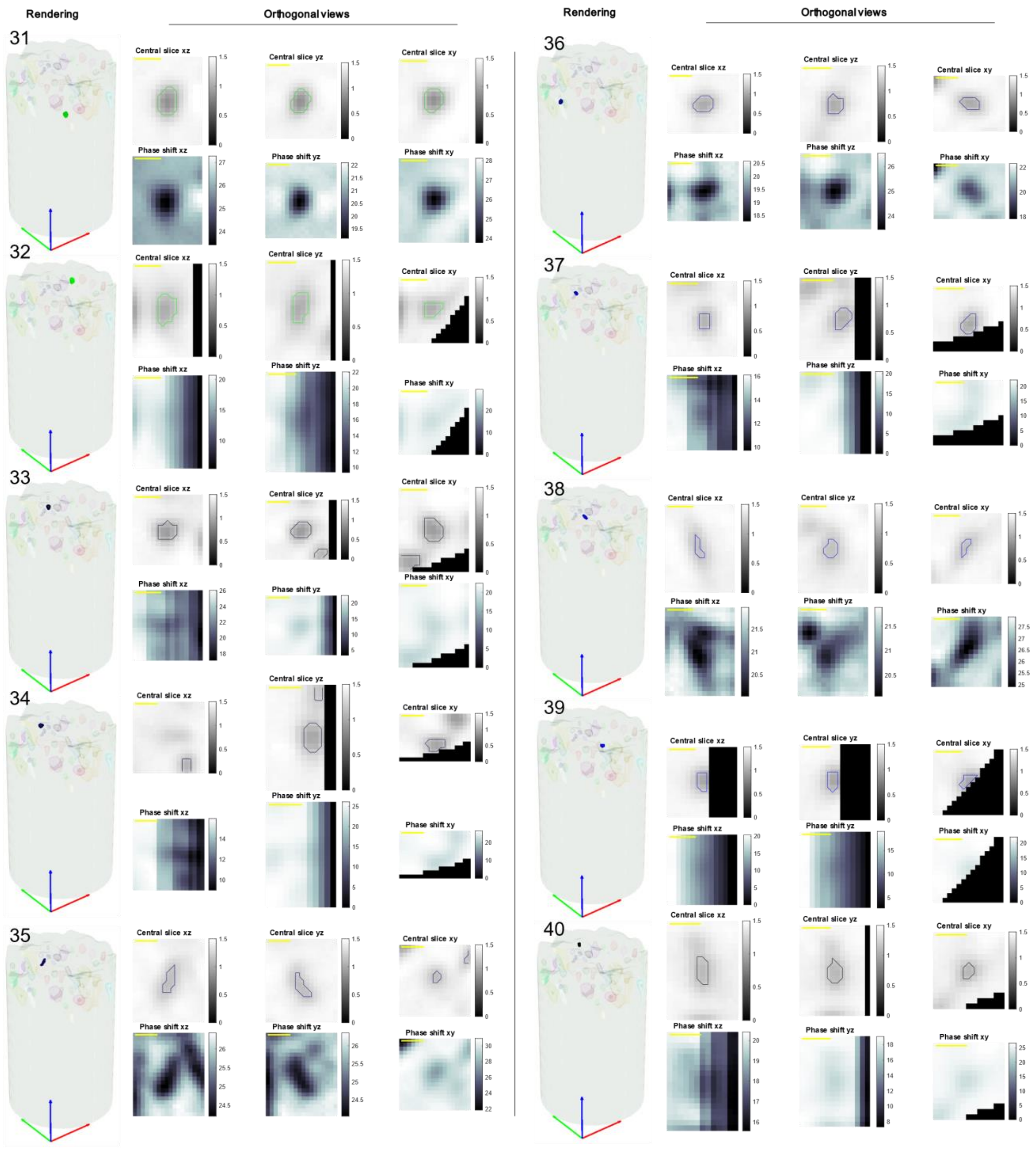

Fig. S10-d. View of single voids.

Each void is represented by label and rendering, orthogonal central slice including segmentation contours (top 3 images, 'gray' colormap) and projection (phase shifts, bottom 3 images, 'bones' colormap). Scale bars are 100 nm, units are electron density for the slices and mrad for phase shifts.

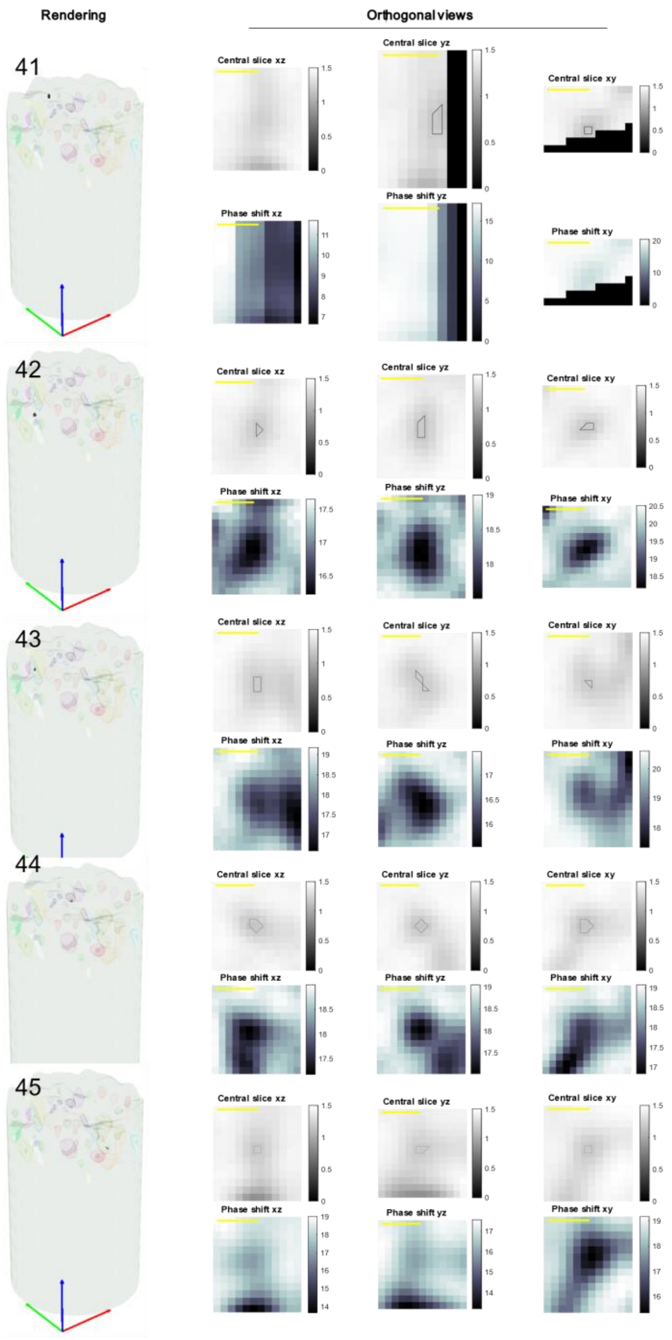

Fig. S10-e. View of single voids.

Each void is represented by label and rendering, orthogonal central slice including segmentation contours (top 3 images, ‘gray’ colormap) and projection (phase shifts, bottom 3 images, ‘bones’ colormap). Scale bars are 100 nm, units are electron density for the slices and mrad for phase shifts.

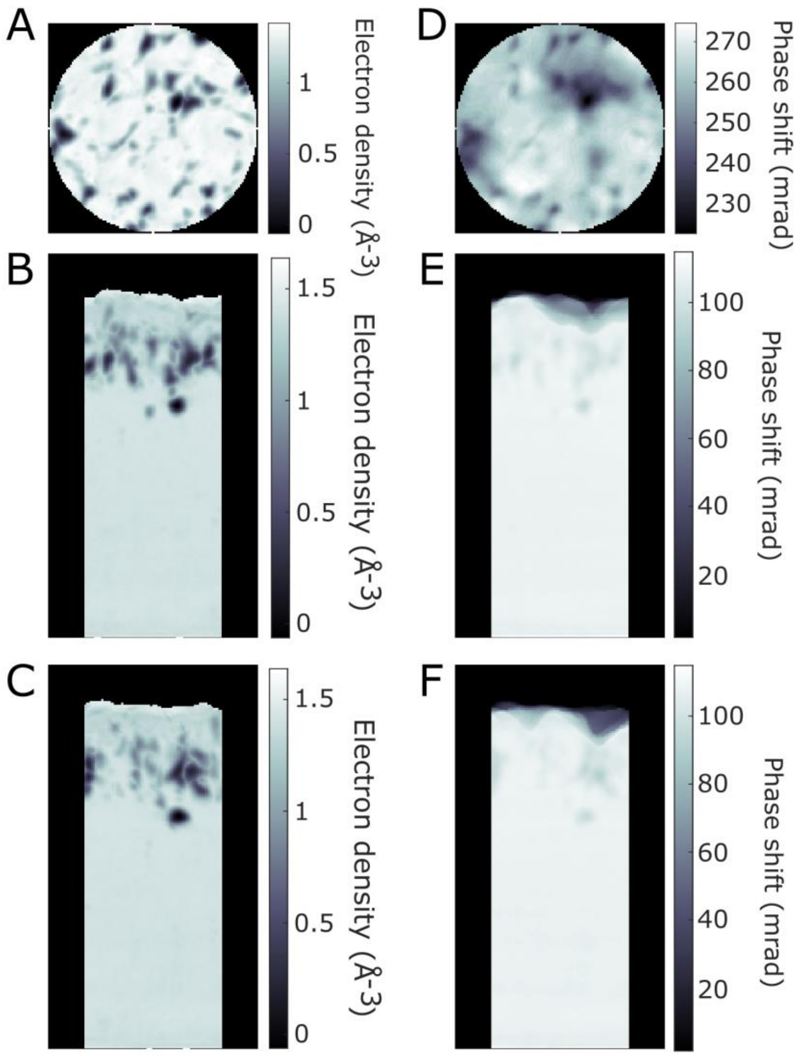

Fig. S11. Comparison of minimum vs sum intensity projections. Orthogonal projections computed from tomography of the 2-μm inner cylinder. (**A**, **B**, **C**) minimum intensity projections. (**D**, **E**, **F**) sum projections. The MgF_2 layer and the lower part of the absorber were excluded for this analysis.

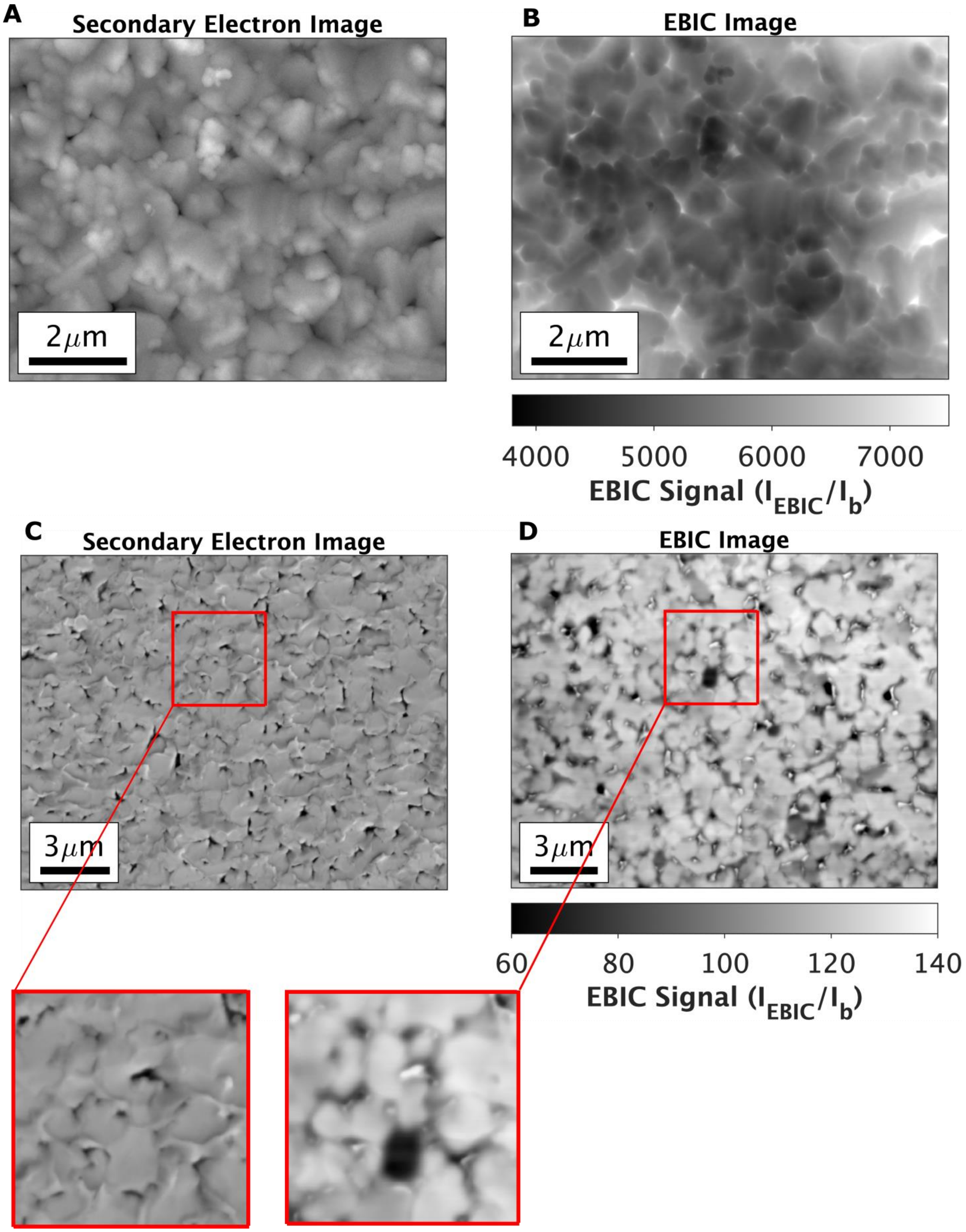

Fig. S12. Scanning electron microscopy (SEM) and electron beam induced current (EBIC) measurements. (A,B) SEM and EBIC images of region 1, before Ar-ion milling. The EBIC intensity follows strictly and inversely the surface topography, being increased at grain boundaries and at their intersections, where voids may be present. (C, D) SEM and EBIC images of the polished region 2. Insets refer to the same 4 x 4 um^2 area.

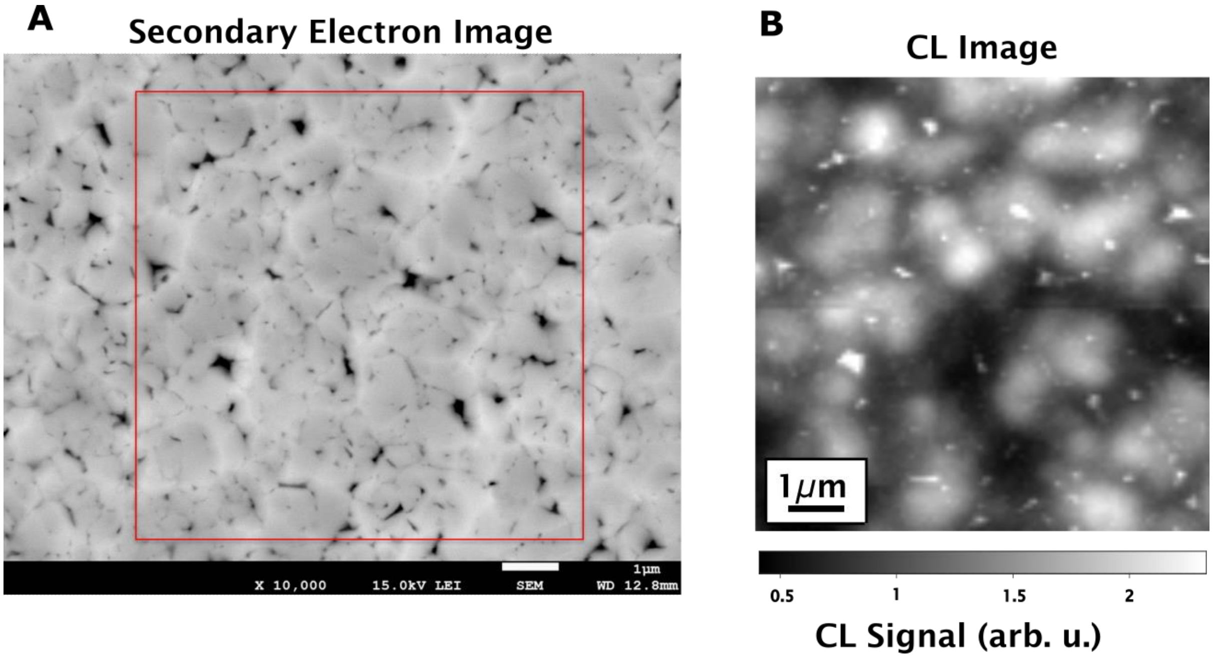

Fig. S13. Scanning electron microscopy (SEM) and cathodoluminescence (CL) images. (A, B) SEM and CL image of polished region 3. The red square in (A) denotes the area imaged through CL. (A) and (B) have the same scale. CL images mimic the grain structure of the absorber. CL spikes occur at SEM dips.

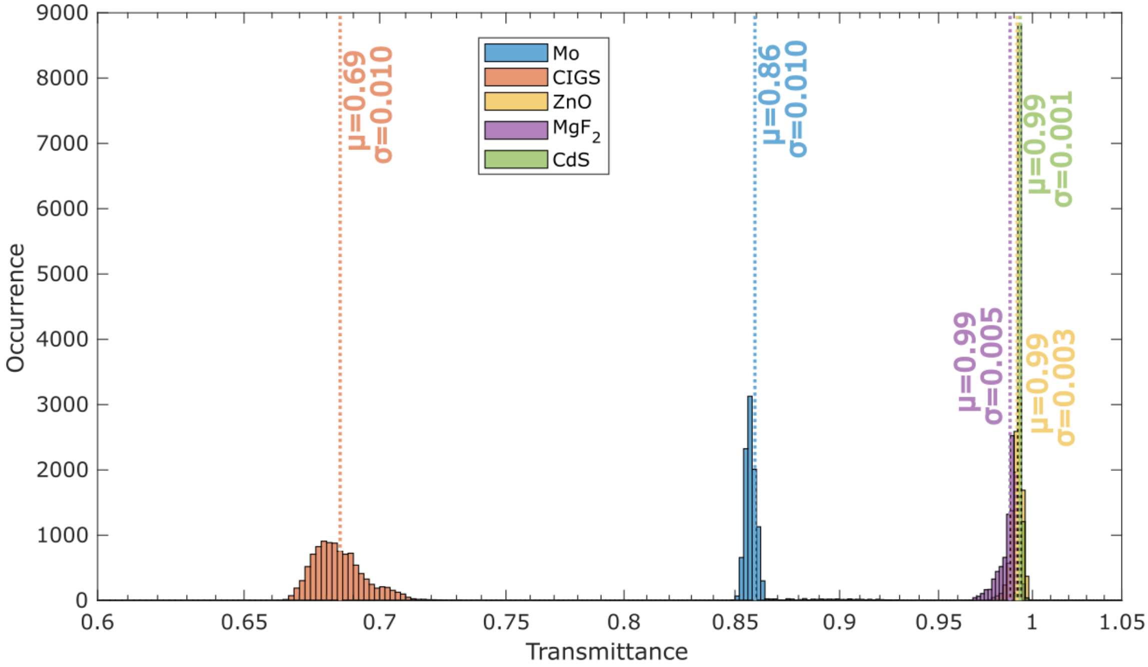

Fig. S14. Transmittance of individual layers extracted from PXCT. Histograms show the occurrence of voxels for different transmittance values; μ and σ denote mean and standard deviation of each distribution.

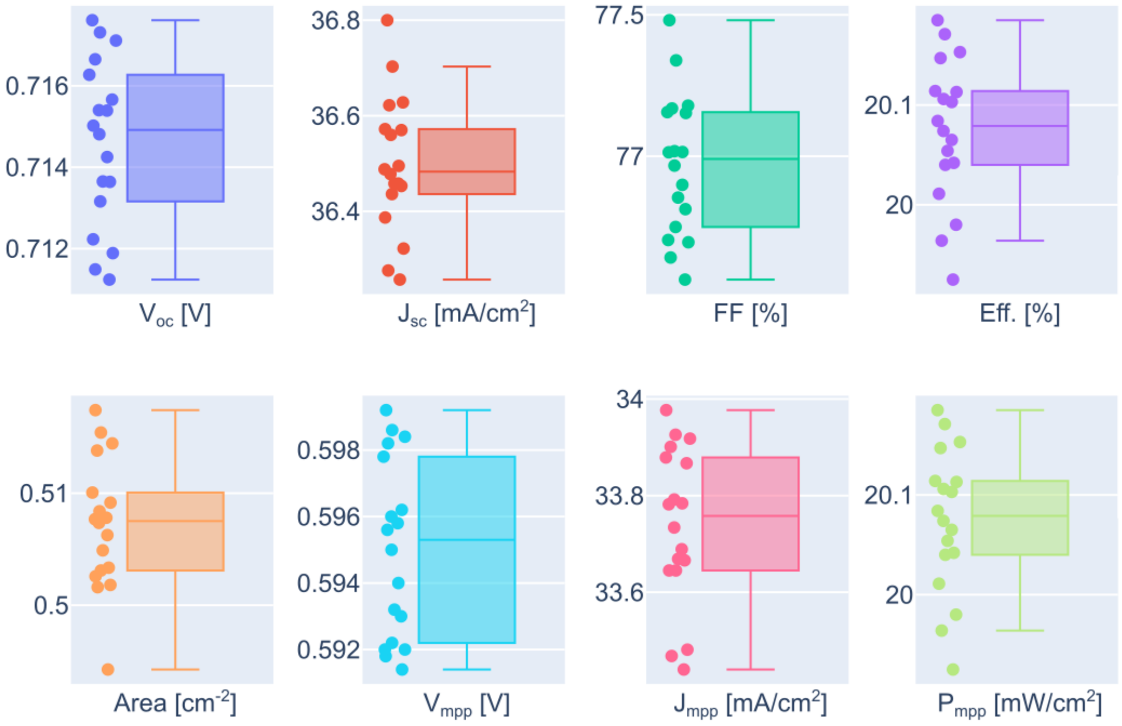

Fig. S15. Box plots of photovoltaic performance parameters for 18 cells of same process.

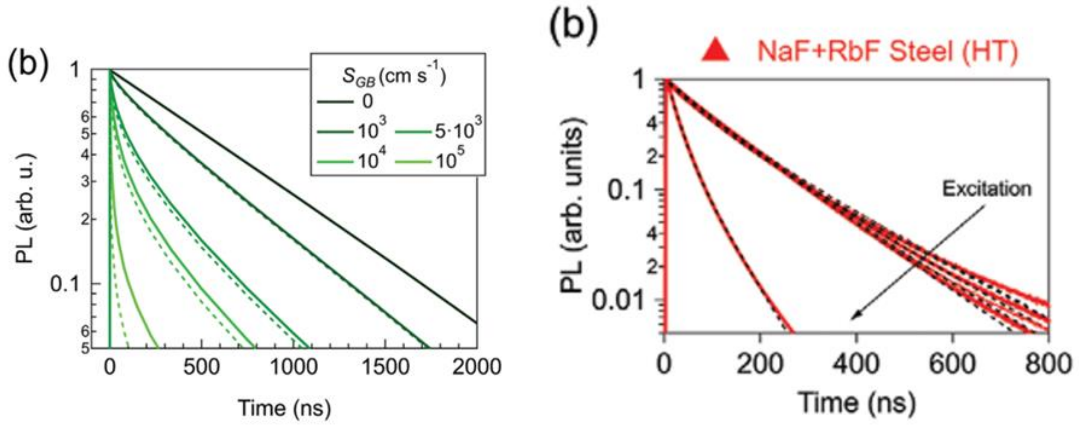

Fig. S16. Extracts from Fig. 3 and Fig. 5 of reference (*30*), reproduced with permission. Left: Simulation of the effect of recombination velocity at grain boundaries. Right: Measured TR-PL decays for devices with low recombination velocity.

Table S1. Parameters for multimodal imaging scans. Three scans were optimized for three modalities and XRF was taken at the same time.

Modality	Scan mode	Step size	Dwell time	Chopper	Slit width
**XBIC**	Fly-scan	25 nm	0.01 s	Yes	0.2 mm
**XEOL**	Fly-scan	50 nm	0.5 s	Yes	0.2 mm
**Ptychography**	Step-scan	50 nm	0.5 s	No	0.04 mm

Table S2. Expected values for refractive indices.

Computed from tabulated values of scattering factors, as described above.

**Materials**	Density (g/cm^3)	$\boldsymbol{n_e}$ (Å^{-3})	$\boldsymbol{\delta}$ (1)	$\boldsymbol{\beta}$ (1)
**Mo**	10.22	2.70	4.83E-05	4.91E-06
**$CuInSe_2$**	5.77	1.48	2.65E-05	2.28E-06
**$CuGaSe_2$**	5.56	1.44	2.58E-05	1.19E-06
**CdS**	4.82	1.28	2.30E-05	2.90E-06
**ZnO**	5.61	1.54	2.77E-05	8.45E-07
**MgF_2**	3.15	0.93	1.66E-05	2.60E-07

Table S3. Correlation sets of layers of Fig. 5.

Computed with α=0.05. The correlation coefficient matrix is referred to as R and the boundaries of 95% confidence intervals are referred to as low-R and high-R.

R	Mo-PI	CIGS	CdS-ZnO-MgF2	Layer stack
Mo-PI	1.00	-0.01	-0.03	0.03
CIGS	-0.01	1.00	-0.67	0.95
CdS-ZnO-MgF2	-0.03	-0.67	1.00	-0.42
Layer stack	0.03	0.95	-0.42	1.00
p-value	Mo-PI	CIGS	CdS-ZnO-MgF2	Layer stack
Mo-PI	1.00	0.49	< 0.01	0.01
CIGS	0.49	1.00	< 0.01	< 0.01
CdS-ZnO-MgF2	< 0.01	< 0.01	1.00	<0.01
Layer stack	0.01	< 0.01	< 0.01	1.00
lower-R	Mo-PI	CIGS	CdS-ZnO-MgF2	Layer stack
Mo-PI	1.00	-0.03	-0.05	0.01
CIGS	-0.03	1.00	-0.69	0.95
CdS-ZnO-MgF2	-0.05	-0.69	1.00	-0.43
Layer stack	0.01	0.95	-0.43	1.00
upper-R	Mo-PI	CIGS	CdS-ZnO-MgF2	Layer stack
Mo-PI	1.00	0.01	-0.01	0.05
CIGS	0.01	1.00	-0.66	0.95
CdS-ZnO-MgF2	-0.01	-0.66	1.00	-0.40
Layer stack	0.05	0.95	-0.40	1.00

Movie S1.

Shows an overview of the layer segmentation, vertical slices, horizontal slices, and volume rendering of voids.

Movie S2.

Shows the orientation of segmented voids. Orientation is defined by the three principal axes of fitting ellipsoids.

References and Notes

1. M. Powalla, S. Paetel, D. Hariskos, R. Wuerz, F. Kessler, P. Lechner, W. Wischmann, T. M. Friedlmeier, Advances in Cost-Efficient Thin-Film Photovoltaics Based on Cu(In,Ga)Se2. *Engineering*. **3**, 445–451 (2017).
2. A. Polman, M. Knight, E. C. Garnett, B. Ehrler, W. C. Sinke, Photovoltaic materials: Present efficiencies and future challenges. *Science*. **352**, aad4424 (2016).
3. M. Jošt, L. Kegelmann, L. Korte, S. Albrecht, Monolithic Perovskite Tandem Solar Cells: A Review of the Present Status and Advanced Characterization Methods Toward 30% Efficiency. *Adv. Energy Mater.* **10** (2020), doi:10.1002/aenm.201904102.
4. Q. Han, Y. T. Hsieh, L. Meng, J. L. Wu, P. Sun, E. P. Yao, S. Y. Chang, S. H. Bae, T. Kato, V. Bermudez, Y. Yang, High-performance perovskite/ Cu(In,Ga)Se2 monolithic tandem solar cells. *Science*. **361**, 904–908 (2018).
5. M. A. Green, E. D. Dunlop, J. Hohl-Ebinger, M. Yoshita, N. Kopidakis, K. Bothe, D. Hinken, M. Rauer, X. Hao, Solar cell efficiency tables (Version 60). *Prog. Photovoltaics Res. Appl.* **30**, 687–701 (2022).
6. D. Colombara, K. Conley, M. Malitckaya, H.-P. Komsa, M. J. Puska, The fox and the hound: in-depth and in-grain Na doping and Ga grading in Cu(In,Ga)Se 2 solar cells. *J. Mater. Chem. A* (2020), doi:10.1039/D0TA01103G.
7. R. Carron, S. Nishiwaki, T. Feurer, R. Hertwig, E. Avancini, J. Löckinger, S.-C. C. Yang, S. Buecheler, A. N. Tiwari, Advanced Alkali Treatments for High-Efficiency Cu(In,Ga)Se2 Solar Cells on Flexible Substrates. *Adv. Energy Mater.* **9**, 1900408 (2019).
8. T. Baines, L. Bowen, B. G. Mendis, J. D. Major, Microscopic Analysis of Interdiffusion and Void Formation in CdTe(1- x)Se xand CdTe Layers. *ACS Appl. Mater. Interfaces*. **12**, 38070–38075 (2020).
9. T. Gershon, K. Sardashti, O. Gunawan, R. Mankad, S. Singh, Y. S. Lee, J. A. Ott, A. Kummel, R. Haight, Photovoltaic Device with over 5% Efficiency Based on an n-Type Ag_2 $ZnSnSe_4$ Absorber. *Adv. Energy Mater.* **6**, 1601182 (2016).
10. G. Seo, D. D. D. Lee, S. Heo, M. Seol, Y. Lee, K. Kim, S. H. Kim, J. J. Lee, D. D. D. Lee, J. J. Lee, D. W. Kwak, D. D. D. Lee, H. Y. Cho, J. Park, T. K. Ahn, M. K. Nazeeruddin, Microscopic Analysis of Inherent Void Passivation in Perovskite Solar Cells. *ACS Energy Lett.* **2**, 1705–1710 (2017).
11. S. Essig, S. Paetel, T. M. Friedlmeier, M. Powalla, Challenges in the deposition of (Ag,Cu)(In,Ga)Se2 absorber layers for thin-film solar cells. *JPhys Mater.* **4** (2021), doi:10.1088/2515-7639/abd73d.
12. Z. Dai, S. K. Yadavalli, M. Chen, A. Abbaspourtamijani, Y. Qi, N. P. Padture, Interfacial toughening with self-assembled monolayers enhances perovskite solar cell reliability. *Science*. **372**, 618–622 (2021).
13. B. J. Stanbery, D. Abou-Ras, A. Yamada, L. Mansfield, CIGS photovoltaics: reviewing an evolving paradigm. *J. Phys. D. Appl. Phys.* **55**, 173001 (2021).
14. E. Avancini, D. Keller, R. Carron, Y. Arroyo-Rojas Dasilva, R. Erni, A. Priebe, S. Di Napoli, M. Carrisi, G. Sozzi, R. Menozzi, F. Fu, S. Buecheler, A. N. Tiwari, Voids and

compositional inhomogeneities in Cu(In,Ga)Se 2 thin films: evolution during growth and impact on solar cell performance. *Sci. Technol. Adv. Mater.* **19**, 871–882 (2018).

15. C. Ossig, T. Nietzold, B. West, M. Bertoni, G. Falkenberg, C. G. Schroer, M. E. Stuckelberger, X-ray Beam Induced Current Measurements for Multi-Modal X-ray Microscopy of Solar Cells. *J. Vis. Exp.* **150**, e60001 (2019).

16. M. E. Stuckelberger, T. Nietzold, B. M. West, B. Lai, J. M. Maser, V. Rose, M. I. Bertoni, in *2017 IEEE 44th Photovoltaic Specialist Conference (PVSC)* (IEEE, 2017; https://ieeexplore.ieee.org/document/8366368/), pp. 2179–2184.

17. C. Ossig, C. Strelow, J. Flügge, A. Kolditz, J. Siebels, J. Garrevoet, K. Spiers, M. Seyrich, D. Brückner, N. Pyrlik, J. Hagemann, F. Seiboth, A. Schropp, R. Carron, G. Falkenberg, A. Mews, C. Schroer, T. Kipp, M. Stuckelberger, Four-Fold Multi-Modal X-ray Microscopy Measurements of a Cu(In,Ga)Se2 Solar Cell. *Materials (Basel).* **14**, 228 (2021).

18. P. Thibault, M. Dierolf, A. Menzel, O. Bunk, C. David, F. Pfeiffer, High-Resolution Scanning X-ray Diffraction Microscopy. *Science.* **321**, 379–382 (2008).

19. A. Saadaldin, A. M. Slyamov, M. E. Stuckelberger, P. S. Jørgensen, C. Rein, M. M. Lucas, T. Ramos, A. Rodriguez-Fernandez, D. Bernard, J. W. Andreasen, Multi-modal characterization of kesterite thin-film solar cells: experimental results and numerical interpretation (2022), doi:10.1039/d2fd00044j.

20. M. Dierolf, A. Menzel, P. Thibault, P. Schneider, C. M. Kewish, R. Wepf, O. Bunk, F. Pfeiffer, Ptychographic X-ray computed tomography at the nanoscale. *Nature*. **467**, 436–439 (2010).

21. M. Holler, A. Diaz, M. Guizar-Sicairos, P. Karvinen, E. Färm, E. Härkönen, M. Ritala, A. Menzel, J. Raabe, O. Bunk, X-ray ptychographic computed tomography at 16 nm isotropic 3D resolution. *Sci. Rep.* **4**, 1–5 (2014).

22. H. F. Dam, T. R. Andersen, E. B. L. Pedersen, K. T. S. Thydén, M. Helgesen, J. E. Carlé, P. S. Jørgensen, J. Reinhardt, R. R. Søndergaard, M. Jørgensen, E. Bundgaard, F. C. Krebs, J. W. Andreasen, Enabling Flexible Polymer Tandem Solar Cells by 3D Ptychographic Imaging. *Adv. Energy Mater.* **5**, 1400736 (2015).

23. G. Falkenberg, F. Seiboth, F. Koch, K. V. Falch, A. Schropp, D. Brückner, J. Garrevoet, CRL optics and silicon drift detector for P06 Microprobe experiments at 35 keV. *Powder Diffr.* **35**, S34–S37 (2020).

24. F. Seiboth, F. Wittwer, M. Scholz, M. Kahnt, M. Seyrich, A. Schropp, U. Wagner, C. Rau, J. Garrevoet, G. Falkenberg, C. G. Schroer, Nanofocusing with aberration-corrected rotationally parabolic refractive X-ray lenses. *urn:issn:1600-5775*. **25**, 108–115 (2018).

25. M. Holler, J. Raabe, A. Diaz, M. Guizar-Sicairos, C. Quitmann, A. Menzel, O. Bunk, An instrument for 3D x-ray nano-imaging. *Rev. Sci. Instrum.* **83**, 073703 (2012).

26. M. Holler, A. Diaz, M. Guizar-Sicairos, P. Karvinen, E. Färm, E. Härkönen, M. Ritala, A. Menzel, J. Raabe, O. Bunk, X-ray ptychographic computed tomography at 16 nm isotropic 3D resolution. *Sci. Reports 2014 41*. **4**, 1–5 (2014).

27. M. Van Heel, M. Schatz, Fourier shell correlation threshold criteria. *J. Struct. Biol.* **151**, 250–262 (2005).

28. T. P. Weiss, F. Ehre, V. Serrano-Escalante, T. Wang, S. Siebentritt, Understanding Performance Limitations of Cu(In,Ga)Se2 Solar Cells due to Metastable Defects—A Route toward Higher Efficiencies. *Sol. RRL*. **5**, 2100063 (2021).

29. S. Siebentritt, T. P. Weiss, M. Sood, M. H. Wolter, A. Lomuscio, O. Ramirez, How photoluminescence can predict the efficiency of solar cells. *J. Phys. Mater.* **4**, 042010 (2021).

30. M. Ochoa, S. C. Yang, S. Nishiwaki, A. N. Tiwari, R. Carron, Charge Carrier Lifetime Fluctuations and Performance Evaluation of Cu(In,Ga)Se2 Absorbers via Time-Resolved-Photoluminescence Microscopy. *Adv. Energy Mater.* **12** (2022), doi:10.1002/aenm.202102800.

31. M. Richter, I. Riedel, C. Schubbert, P. Eraerds, J. Parisi, T. Dalibor, J. Palm, Simulation study of the impact of interface roughness and void inclusions on Cu(In,Ga)(Se,S)2 solar cells. *Phys. Status Solidi Appl. Mater. Sci.* **212**, 298–306 (2015).

32. O. Lundberg, M. Edoff, L. Stolt, in *Thin Solid Films* (Elsevier, 2005), vols. 480–481, pp. 520–525.

33. A. Diaz, P. Trtik, M. Guizar-Sicairos, A. Menzel, P. Thibault, O. Bunk, Quantitative x-ray phase nanotomography. *Phys. Rev. B*. **85**, 020104 (2012).

34. S. Yang, M. Ochoa, R. Hertwig, A. Aribia, A. N. Tiwari, R. Carron, Influence of Ga back grading on voltage loss in low-temperature co-evaporated Cu(In,Ga)Se2 thin film solar cells. *Prog. Photovoltaics Res. Appl.* **29**, 630–637 (2021).

35. P. Schöppe, S. Schönherr, R. Wuerz, W. Wisniewski, G. Martínez-Criado, M. Ritzer, K. Ritter, C. Ronning, C. S. Schnohr, Rubidium segregation at random grain boundaries in Cu(In,Ga)Se2 absorbers. *Nano Energy*. **42**, 307–313 (2017).

36. D. L. McGott, C. P. Muzzillo, C. L. Perkins, J. J. Berry, K. Zhu, J. N. Duenow, E. Colegrove, C. A. Wolden, M. O. Reese, 3D/2D passivation as a secret to success for polycrystalline thin-film solar cells. *Joule*. **5**, 1057–1073 (2021).

37. C. T. Plass, M. Ritzer, P. Schöppe, S. Schönherr, M. Zapf, M. Hafermann, A. Johannes, G. Martínez-Criado, J. Segura-Ruiz, R. Würz, P. Jackson, C. S. Schnohr, C. Ronning, In-Operando Nanoscale X-ray Analysis Revealing the Local Electrical Properties of Rubidium-Enriched Grain Boundaries in Cu(In,Ga)Se 2 Solar Cells. *ACS Appl. Mater. Interfaces*. **12**, 57117–57123 (2020).

38. P. Schöppe, S. Schönherr, P. Jackson, R. Wuerz, W. Wisniewski, M. Ritzer, M. Zapf, A. Johannes, C. S. Schnohr, C. Ronning, Overall Distribution of Rubidium in Highly Efficient Cu(In,Ga)Se 2 Solar Cells. *ACS Appl. Mater. Interfaces*. **10**, 40592–40598 (2018).

39. N. Nicoara, R. Manaligod, P. Jackson, D. Hariskos, W. Witte, G. Sozzi, R. Menozzi, S. Sadewasser, Direct evidence for grain boundary passivation in Cu(In,Ga)Se2 solar cells through alkali-fluoride post-deposition treatments. *Nat. Commun.* **10**, 3980 (2019).

40. D. D. Gürsoy, C. Jacobsen, Multimodal x-ray nanotomography. *MRS Bull.* **45**, 272–276 (2020).

41. J. Deng, Y. H. Lo, M. Gallagher-Jones, S. Chen, A. Pryor, Q. Jin, Y. P. Hong, Y. S. G. Nashed, S. Vogt, J. Miao, C. Jacobsen, Correlative 3D x-ray fluorescence and

ptychographic tomography of frozen-hydrated green algae. *Sci. Adv.* **4**, eaau4548 (2018).

42. S. Siebentritt, E. Avancini, M. Bär, J. Bombsch, E. Bourgeois, S. Buecheler, R. Carron, C. Castro, S. Duguay, R. Félix, E. Handick, D. Hariskos, V. Havu, P. Jackson, H. P. Komsa, T. Kunze, M. Malitckaya, R. Menozzi, M. Nesladek, N. Nicoara, M. Puska, M. Raghuwanshi, P. Pareige, S. Sadewasser, G. Sozzi, A. N. Tiwari, S. Ueda, A. Vilalta-Clemente, T. P. Weiss, F. Werner, R. G. Wilks, W. Witte, M. H. Wolter, Heavy Alkali Treatment of Cu(In,Ga)Se2 Solar Cells: Surface versus Bulk Effects. *Adv. Energy Mater.* **10**, 1903752 (2020).

43. M. Holler, M. Odstrcil, M. Guizar-Sicairos, M. Lebugle, E. Müller, S. Finizio, G. Tinti, C. David, J. Zusman, W. Unglaub, O. Bunk, J. Raabe, A. F. J. Levi, G. Aeppli, Three-dimensional imaging of integrated circuits with macro- to nanoscale zoom. *Nat. Electron.* **2**, 464–470 (2019).

44. M. E. Stuckelberger, T. Nietzold, B. West, R. Farshchi, D. Poplavskyy, J. Bailey, B. Lai, J. M. Maser, M. I. Bertoni, Defect activation and annihilation in CIGS solar cells: an operando x-ray microscopy study. *J. Phys. Energy*. **2**, 025001 (2020).

45. D. Colombara, H. Elanzeery, N. Nicoara, D. Sharma, M. Claro, T. Schwarz, A. Koprek, M. H. Wolter, M. Melchiorre, M. Sood, N. Valle, O. Bondarchuk, F. Babbe, C. Spindler, O. Cojocaru-Miredin, D. Raabe, P. J. Dale, S. Sadewasser, S. Siebentritt, Chemical instability at chalcogenide surfaces impacts chalcopyrite devices well beyond the surface. *Nat. Commun. 2020 111*. **11**, 1–14 (2020).

46. R. Hettel, DLSR design and plans: an international overview. *J. Synchrotron Radiat.* **21**, 843–855 (2014).

47. M. Odstrčil, M. Lebugle, M. Guizar-Sicairos, C. David, M. Holler, Towards optimized illumination for high-resolution ptychography. *Opt. Express*. **27**, 14981 (2019).

48. K. Wakonig, H.-C. Stadler, M. Odstrčil, E. H. R. Tsai, A. Diaz, M. Holler, I. Usov, J. Raabe, A. Menzel, M. Guizar-Sicairos, PtychoShelves , a versatile high-level framework for high-performance analysis of ptychographic data. *J. Appl. Crystallogr.* **53**, 574–586 (2020).

49. I. Johnson, A. Bergamaschi, H. Billich, S. Cartier, R. Dinapoli, D. Greiffenberg, M. Guizar-Sicairos, B. Henrich, J. Jungmann, D. Mezza, A. Mozzanica, B. Schmitt, X. Shi, G. Tinti, Eiger: a single-photon counting x-ray detector. *J. Instrum.* **9**, C05032–C05032 (2014).

50. P. Thibault, M. Guizar-Sicairos, Maximum-likelihood refinement for coherent diffractive imaging. *New J. Phys.* **14**, 063004 (2012).

51. M. Guizar-Sicairos, J. J. Boon, K. Mader, A. Diaz, A. Menzel, O. Bunk, Quantitative interior x-ray nanotomography by a hybrid imaging technique. *Optica*. **2**, 259 (2015).

52. M. Guizar-Sicairos, A. Diaz, M. Holler, M. S. Lucas, A. Menzel, R. A. Wepf, O. Bunk, Phase tomography from x-ray coherent diffractive imaging projections. *Opt. Express*. **19**, 21345 (2011).

53. T. J. Cleophas, A. H. Zwinderman, *Modern Meta-Analysis* (Springer International Publishing, Cham, 2017; http://link.springer.com/10.1007/978-3-319-55895-0).

54. C. Ossig, N. Pyrlik, R. Carron, G. Fevola, S. Patjens, C. Strelow, J. Flügge, A. Kolditz, J.

Siebels, J. Garrevoet, K. Spiers, M. Seyrich, D. Brückner, J. Hagemann, F. Seiboth, A. Schropp, G. Falkenberg, A. Mews, C. G. Schroer, T. Kipp, M. E. Stuckelberger, X-ray vision of Cu(In,Ga)Se 2 : from the Ga/In ratio to solar-cell performance. *J. Phys. Energy*. **4**, 045007 (2022).

55. S. Sasaki, "Numerical Tables of Anomalous Scattering Factors Calculated by the Cromer and Liberman's Method" (Tsukuba, 1989), (available at https://inis.iaea.org/search/search.aspx?orig_q=RN:20081580).

56. G. Fevola, P. S. Jørgensen, M. Verezhak, A. Slyamov, A. Crovetto, Z. I. Balogh, C. Rein, S. Canulescu, J. W. Andreasen, Resonant x-ray ptychographic nanotomography of kesterite solar cells. *Phys. Rev. Res.* **2**, 013378 (2020).

57. B. G. Mendis, L. Bowen, Q. Z. Jiang, A contactless method for measuring the recombination velocity of an individual grain boundary in thin-film photovoltaics. *Appl. Phys. Lett.* **97** (2010), doi:10.1063/1.3486482.

58. M. Krause, A. Nikolaeva, M. Maiberg, P. Jackson, D. Hariskos, W. Witte, J. A. Márquez, S. Levcenko, T. Unold, R. Scheer, D. Abou-Ras, Microscopic origins of performance losses in highly efficient Cu(In,Ga)Se2 thin-film solar cells. *Nat. Commun.* **11** (2020), doi:10.1038/s41467-020-17507-8.

59. S. Koho, G. Tortarolo, M. Castello, T. Deguchi, A. Diaspro, G. Vicidomini, Fourier ring correlation simplifies image restoration in fluorescence microscopy. *Nat. Commun. 2019 101*. **10**, 1–9 (2019).

Parts of this research were carried out at beamline P06 of PETRA III at Deutsches Elektronen-Synchrotron DESY, a member of the Helmholtz Association (HGF). This research was supported in part through the Maxwell computational resources operated at Deutsches Elektronen-Synchrotron DESY.

We thank cSAXS beamline, Paul Scherrer Institute, Switzerland for providing beamtime. M.V. acknowledges financial support from the European Union's Horizon 2020 research and innovation program under the Marie Skłodowska-Curie Grant Agreements No. 701647 (M.V.)

This work was authored in part by the National Renewable Energy Laboratory, operated by Alliance for Sustainable Energy, LLC, for the U.S. Department of Energy (DOE) under Contract No. DE-AC36-08GO28308. Funding provided by U.S. Department of Energy Office of Energy Efficiency and Renewable Energy Solar Energy Technologies. The views expressed in the article do not necessarily represent the views of the DOE or the U.S. Government. The U.S. Government retains and the publisher, by accepting the article for publication, acknowledges that the U.S. Government retains a nonexclusive, paid-up, irrevocable, worldwide license to publish or reproduce the published form of this work, or allow others to do so, for U.S. Government purposes.

J.W.A. acknowledges the H2020 European Research Council through the SEEWHI Consolidator grant, ERC-2015-CoG-681881 and H2020 research and innovation programme under the Marie Skłodowska-Curie grant agreement No. 765604 (MUMMERING)."
